# Worldwide flavor enhancer monosodium glutamate combined with high lipid diet provokes metabolic alterations and systemic anomalies: An overview

**DOI:** 10.1016/j.toxrep.2021.04.009

**Published:** 2021-04-29

**Authors:** Arnab Banerjee, Sandip Mukherjee, Bithin Kumar Maji

**Affiliations:** Department of Physiology (UG & PG), Serampore College, 9 William Carey Road, Serampore, Hooghly, 712201, West Bengal, India

**Keywords:** Food habits, Monosodium glutamate, High lipid diet, Human health, Harmful effects

## Abstract

In this fast-food era, people depend on ready-made foods and engage in minimal physical activities that ultimately change their food habits. Majorities of such foods have harmful effects on human health due to higher percentages of saturated fatty acids, trans-fatty acids, and hydrogenated fats in the form of high lipid diet (HLD). Moreover, food manufacturers add monosodium glutamate (MSG) to enhance the taste and palatability of the HLD. Both MSG and HLD induce the generation of reactive oxygen species (ROS) and thereby alter the redox-homeostasis to cause systemic damage. However, MSG mixed HLD (MH) consumption leads to dyslipidemia, silently develops non-alcoholic fatty liver disease followed by metabolic alterations and systemic anomalies, even malignancies, via modulating different signaling pathways. This comprehensive review formulates health care strategies to create global awareness about the harmful impact of MH on the human body and recommends the daily consumption of more natural foods rich in antioxidants instead of toxic ingredients to counterbalance the MH-induced systemic anomalies.

## Highlights

• Flavor enhancing high lipid diet acts as silent killer.
• Monosodium glutamate mixed with high lipid diet alters redox-status.
• Monosodium glutamate mixed with high lipid diet induces systemic anomalies.

## Graphical abstract

**Figure:**
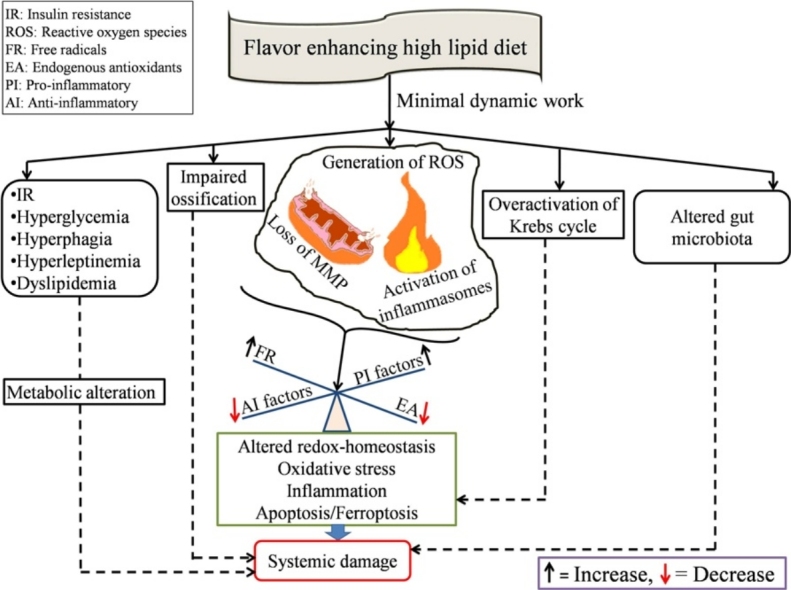

## 1 Introduction

Taste perception plays a vital role in regulating food preference, eating patterns, and homeostatic energy balance control. Various factors modulate the gustation [1]. All over the world, unnecessary modification of food habits and delicious food are directly associated with general people’s health issues. Nowadays, people are busy with their technology-based busy work schedules in different sectors, industries, and companies. In such a fast-paced life, they have very minimal time preparing their meals and engaging in physical activities [2]. For these reasons, most of them are dependent on “ready-made foods” or “junk foods” either from the canteen or nearby cafeterias or street-side local restaurants or Dhaba. Such habits ultimately change their food habits. Most of the “ready-made foods” contain high amounts of saturated fatty acids like palmitic acid, lauric acid, myristic acid. Thus, in combination with trans-fatty acids and hydrogenated fats, oxidative stress (OS) can result in inflammation to alter the redox-status and apoptosis, followed by metabolic disorders and systemic damage [[3], [4], [5]]. Such foods are preferred by all age groups because of their taste, effortless preparation, ease of consumption. These foods have saturated fatty acids and hydrogenated fats, which prevent the foods from rancidity without refrigeration. Such foods are considered unhealthy because of low nutritional value, and it is also known as junk food [[6], [7], [8]]. However, to increase taste and palatability, food producers use monosodium glutamate (MSG) [9]. This savory taste sensation of MSG escalates the attraction towards the specific type of foods or food products. As per the earlier survey report, globally, Asia is the stockpile of MSG production. Moreover, the consumption of MSG in the diet is higher in the Middle East and Africa than in Europe, North America, Central and South America [10]. Besides using MSG in junk food or ready-made foods, MSG is present in a wide variety of protein-rich foods like Roquefort, meat, Parmesan, fish, mushrooms, broccoli, tomatoes, etc. Additionally, an earlier study suggested that the human body cannot differentiate added MSG from naturally occurring glutamate in the diet. Moreover, umami taste sensation is also highly found in protein-rich foods [11]. On the other hand, saturated fats are present in protein-rich food like hamburgers, and fried chicken, which is also considered junk food [12]. Frozen entrees, canned tuna, crackers, processed meats, soups, dietary supplements, cosmetics, salad dressings, infant formula, vaccines, and other food products also contain MSG as a food seasoning chemical. MSG also applies to process the free glutamic acid that has been freed from protein through a manufacturing process of different cosmetics and other products. Moreover, in vaccines MSG has also been used as as preservative and stabilizer, to keep the vaccines effective through temperature and shelf life [10]. Ready-made foods or junk foods become more and more popular among people with the arrival of pizza, hamburgers, fried chicken, chips, hot dog, pakora, Chinese stir-fried noodles, French fries, cheese chili, pav bhaji, momo, and some other snacks and “spicy yummy” food items either from local restaurants or cafeteria [3,8,12]. Previously, researchers proposed that there is a positive relationship between fast-food restaurants and obesity [3]. Based on a survey report by the Institute of Food Technologists, about 75 % of Americans are dependent on 50 % of fast-food from local restaurants during meals, including dinner time [6]. Another study showed a positive correlation between metabolic disorders and MSG among Thailand’s rural people [13]. Nowadays, there has been a radical change in food habits in the USA, Canada, Britain, Australia, Japan, Sweden, and India [6,12]. About 40 % of the industries are concerned with the preparation of fast-food per year in India. Every year 2.1 % of India's total expenditure is spent on fast-food, and India ranks tenth in statistics. An earlier study showed that ready-made foods with a lack of physical activities could harm the lives of many people worldwide in the next coming ten years [6]. According to the demand of the people worldwide, fast-food restaurants, cafeterias, and the chain are increasing rapidly [12]. It ultimately leads to an increased risk of obesity, dyslipidemia, diabetes, metabolic disorders, non-alcoholic fatty liver diseases, steatosis of the liver, cardiovascular disease, and many other chronic health problems [3]. Western dietary patterns with consumption of ready-made foods regularly are the global burden of different metabolic disorders. The present study aims to review the current evidence about MSG mixed HLD (MH) adverse effects on human health. A recent study by Banerjee et al. [14] stated that MSG mixed HLD provokes serious hepato-cardiac anomalies by altering redox-equilibrium, inflammatory response, and programmed cell death, activating NF-kB and mitochondrial caspase-mediated pathway [14]. To confirm the relation between inflammation, redox imbalance, metabolic disarray, this study was well corroborated with earlier investigations [[15], [16], [17]]. However, there is little information available on chronic human consumption of MH-associated systemic anomalies. Hence, we have attempted to review and understand the possible health impact of such junk foods. It may help formulate health care strategies and create global awareness regarding the harmful effects of regularly using seasoning substances like MSG in HLD.

## 2 Modern diets, food habits, and health problems

In today's world, people devote very little time to prepare their own meals and are mainly dependent on ready-made foods with poor dietetic values that are unhealthy for human beings [8,9]. Over the past few years, regular consumption of chemically processed foods with food additives has remarkably increased in the Western world. This type of food triggered addictiveness towards specific foods [18,19]. Industrially processed foods are responsible for higher intakes of fast-foods per the survey report of national representative INCA3 in France [20]. Globally, there is a positive link between the consumption of such kinds of food and their impact on human health [18]. Fast-foods are made more attractive by adding food additives, synthetic food colors to upheld flavor, texture and by enhancing their long shelf life [21]. Increased shelf life due to the presence of saturated fatty acids and hydrogenated fats, which resists rancidity [3] with various food additives but mostly monosodium glutamate [21]. According to Banerjee et al. (2020), ready-made foods contains a mixture of saturated fats, hydrogenated or partially hydrogenated fats. These foods can stimulate taste and upheld appetite centers with different toxic effects on experimental animals and humans [3]. Subsequently, MSG consumption leads to disruption of energy balance and disturbs the leptin-mediated hypothalamus signaling pathway, leading to obesity [22,23]. Bawaskar et al. [24] demonstrated that consumption of fast-food rich in MSG causes several symptoms like burning sensation in different parts of the body, blistering on arms, occasionally on the anterior thorax, weakness, fatigue, and palpitations just after 20 min of taking meal [24].

**Fig. 1:**
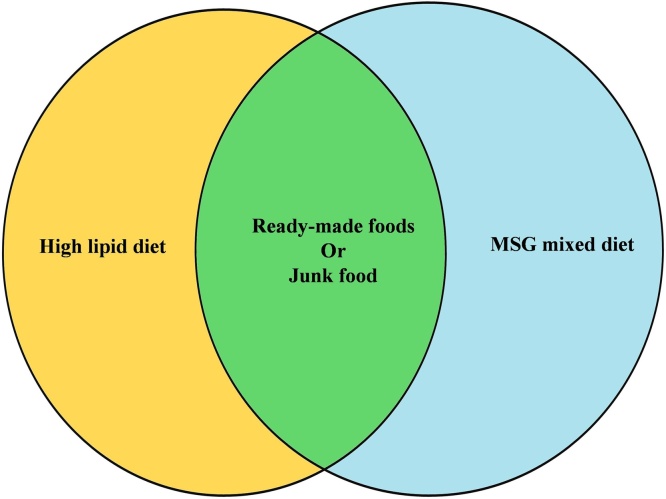
Common factor in ready-made foods or junk food.

Modification or changes in food habits play a pivotal role in developing chronic diseases. Moreover, sedentary behavior and regular consumption of unhealthy fast-foods are other significant factors that stimulate the progression of various chronic diseases such as obesity, cardiovascular disease, diabetes, food poisoning, dehydration, cardiac problems, arthritis, stroke, and cancer [8,25]. Unfortunately, we have adapted to a system of eating foods with several adverse effects on our health. Due to lifestyle changes and busy work schedules, people have minimal time to think about nutrition and fitness. Due to globalization, people have been forced to change their food habits and dietary patterns and consume high-calorie-made fast-foods regularly [21]. Nowadays, regular consumption of such unhealthy foods and their adverse effects on ordinary people become a global health problem. To overcome the issue, proper health education about the hazardous impact of such foods or food products, food habits, and a guideline on healthy eating practice for a better life. Fig. 1 shows the common factor in ready-made foods or junk food.

## 3 MSG, used as a flavor-enhancing agent, causes systemic anomalies

MSG has been used as a flavor-enhancing chemical and an umami tastant [18]. It might have a pathophysiological and toxicological impact on human health [26,27]. Umami’s taste intensifies the meaty, savory flavor of food, just like glutamate does in foods. The universal use of MSG is not less than 100 years for its use as a food seasoning chemical. The metabolism of naturally occurring glutamate occurs in the gut by exopeptidase during hydrolysis of protein in humans [28]. MSG contains hydrolyzed vegetable protein that stimulates orosensory receptors and acts as a flavor enhancer chemical by enhancing foods’ palatability. Excessive use of MSG induces obesity by influencing the appetite center. Earlier reports suggested that very minimal dose (i.e., 0.6 and 1.6 mg/g body weight for two weeks or 100−500 mg/kg body weight for three weeks) of MSG has deleterious effects on human and experimental animals [11,29,30]. Furthermore, it is not easy to conduct an experimental trial to identify the chronic toxicity of MSG in humans as there are several complications, ethical implications, and dietary instructions. But rodent models are very productive models for measuring excitotoxic neurotransmission in mammals [31]. For this reason, the majority of the review articles are on rodent models.

The Food and Drug Administration (FDA) of the United States and European Food Safety Association (EFSA) report that MSG is safe and that it should be maintained on the “Generally Recognized as Safe” (GRAS)-list of foods. Apart from the various food additives used in the preparation of fast-foods, EFSA sets an acceptable daily intake (ADI) of 30 mg/kg/day for universally used flavor-enhancing chemical MSG, while the no-observed-adverse-effect level (NOAEL) has been set at 3200 mg/kg body weight of an individual (Committee on Toxicity of Chemicals in Food, Consumer Products, and the Environment, 2006) [11]. MSG is thus reportedly permitted as a safe food additive that needs no specified average, daily intake, or an upper limit intake requirement [32]. But there are various reports which show the opposite inference from their results. Besides, to investigate the effect of MSG on different animal models, an earlier study showed that the median lethal dose (LD50) of MSG is about 15 g/kg body weight in the murine model. However, it is 18 g/kg body weight, and the LD50 of salt is 3 g/kg body weight in rats which is approximately five folds lower than the LD50 value of MSG [28]. Moreover, out of two isomers of MSG, the first one is l-glutamate and the second one is d-glutamate enantiomer, respectively. l-glutamate enantiomers only exert flavor-enhancing properties. But, various companies prepared MSG with 99.6 % of naturally predominant l-glutamate form to enhance the flavor in fast-food for their marketing purpose, which is greater than the free glutamate ions found in natural foods [33].

Flavor enhancers can offer taste modification in different ready-made foods by using China salt or Ajinomoto. Immense use of MSG leads to systemic damages in different ways. Monosodium glutamate binds to the glutamate receptor and alters the signaling cascade of the hypothalamus. It also disrupts leptin’s action, increases the palatability of food, increases pro-inflammatory cytokines like IL (interleukin)-6 and TNF (tumor necrosis factor)-α, impairs glucose tolerance, increases insulin, leptin, and resistin, respectively. These altered factors ultimately lead to obesity [34]. Insulin resistance (IR) and diabetes have also been associated with the activation of inflammatory mediators like C-reactive protein (CRP), IL-1β, TNF-α and IL-6 in blood and adipose tissue. However, it is speculated that c-Jun N-terminal kinase (JNK)/activator protein 1 (AP1) and IkB kinase (IKK)/nuclear factor kappa-B (NF-kB) might play an essential predominantly role in MSG-induced inflammation by the influence of pro-inflammatory mediators, free fatty acids and ROS. Thus, the production of ROS stimulates adverse effects on the insulin signaling pathway. Moreover, adipose tissue plays a vital role in inflammation and IR in obese conditions via the secretion of specific chemokines, cytokines, and adipocytokines; furthermore, insulin sensitivity is regulated adipocytokines on skeletal muscle and hepatocytes [35].

Monosodium glutamate causes Chinese restaurant syndrome (CRS) by showing some symptoms like back neck burn, blistering on arms, anterior thorax, flushing, dizziness, syncope, and facial pressure, which occur just 20 min after taking a ready-made foods rich in MSG [24]. Neurotoxin releases from the patient with CRS also lead to Alzheimer’s disease, brain tumor, schizophrenia, dementia, anxiety, Tourette syndrome, Huntington disease, multiple sclerosis, Parkinson’s disease, epilepsy, etc. Moreover, the binding of MSG to glutamate receptors also disrupt stromal cells and basement membrane, cellular hypertrophy of the ovarian theca follicular, OS, deoxyribonucleic acid (DNA) damage, protein modification, and some other reproductive abnormalities [34]. Fast-food containing Ajinomoto could produce some alarming symptoms like weakness, flushing, numbness, dizziness, sweating, and headaches, leading to various other complications, including urticaria, asthma, and ventricular arrhythmia, atopic dermatitis, abdominal discomfort, and neuropathy also [36]. Another study proposed that MSG intake is directly associated with higher hemoglobin levels in males than females in the Chinese population. Since leptin plays a vital role in generating cellular components of blood and blood plasma, the effect of MSG on hemoglobin might be accompanied by leptin [15,16,37].

Onakewhor et al. [38] suggested that MSG has a significant toxicological impact on the testis by resulting low count of sperm in the semen and a high count of abnormal sperm in a rat model by dose-dependent manner [38]. Testicular damage, loss of functions, altered structure, and reduction in the population of sperm ultimately lead to male-infertility [39]. Besides, MSG has also cause degeneration of the retina, damage of brain cells, endocrine disorders, and pathological conditions like amyotrophic lateral sclerosis, Huntington’s disease, Parkinson’s disease, Alzheimer’s disease, schizophrenia, anxiety, depression, brain trauma, addiction, neuropathic pain, stroke, etc. [40].

**Fig. 2:**
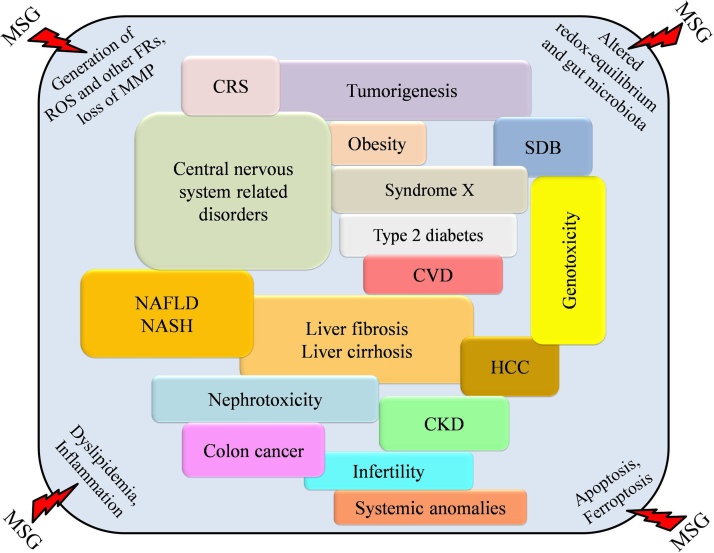
MSG induced metabolic alteration associated deleterious impact on different system.

Glutamate is an excitatory neurotransmitter, and mostly the excitotoxicity is mediated by NMDA receptors as in neuroinflammatory demyelinating and amyotrophic lateral sclerosis. MSG in ready-made foods has also been linked to nephrotoxicity, hepatotoxicity, asthma, urticaria, neoplastic cell growth, and differentiation [41]. MSG as a food additive also causes angioedema and urticaria in randomized controlled trials in humans [36,42]. According to Beyreuther et al. [43], consumption of 16.0 mg/kg of body weight of MSG is considered safe [43]. Nowadays, the application of MSG in different foods was used as compared to the safe level (e.g., 400 mg to 14 g per day) [13]; in the United Kingdom for the general population, the average consumption of MSG was 580 mg per day of an individual and 4.68 g per day for maximal customers [44]. It was reported that every 100 g of Chinese foods contains about 10 mg to 1.5 g of MSG by a joint investigation team of Australia and New Zealand Government in 2003 [45]. Additionally, MSG induced cardiac tissue architecture damage, altered cardiac rhythm associated with biochemical changes, metabolic changes, overweight, liver toxicity, fibrosis, alteration in average growth of cells (i.e., neoplastic), and neuronal damage [11]. Moreover, MSG can alter the behavioral pattern or violent behavior, decreased movements, and reduced muscle strength like a physiological response [46]. Earlier studies also suggested that elevated levels of lipid peroxidation with increased concentration of nitrite and reduced antioxidants levels alter the neuronal redox homeostasis, the hippocampus’s neuronal architecture, increased cholinesterase levels in the brain and serum [47,48]. Fig. 2 shows the overall deleterious impact of MSG.

### 3.1 Effects of MSG on the central nervous system (CNS)

In mammals, excitatory neurotransmitters like glutamate play a significant role in the different processes related to physiology and pathology in the central nervous system [49]. Accumulating evidence suggests that higher aggressiveness is observed in the animals, which could be due to the overactivation of glutamate pathways linked with lower *γ*-aminobutyric acid (GABA) levels for the functions of CNS [46,50], and free radical-mediated dopaminergic neurodegeneration decreases locomotor activity [50]. MSG is also linked with disturbance in the balance between endogenous antioxidant (EA) and free radicals (FRs) and fails to maintain the antioxidant defense homeostasis, loss of membrane integrity and function of neurons, intensified different ions permeability nonspecifically, and pathological alterations of intracellular metabolic pathways [50]. Additionally, MSG has also been linked with cell death, decreased photoreceptor and glial cells in infant mammals’ neurons [51]. On the other hand, MSG incites flavor-preference learning by activating celiac and gastric branches of the 10th cranial nerve to further activate the insula and insular lobe, hypothalamus, limbic system, and nucleus tractus solitarius [52]. We suggest that the research should focus on the impact of MSG on CNS with the gastrointestinal signaling cascade.

Besides, MSG has been associated with several modifications in the hippocampus by downregulation of cyclic 5′ adenosine monophosphate-activated protein kinase (AMPK) and upregulation of pro-apoptotic Fas ligand, Bax [48,53]. Moreover, MSG also altered the histomorphology of hippocampal neuronal damage in the cerebral cortex and cerebellum in the rodent model [47,50]. Onaolapo et al. [47] showed that total brain glutamate or glutamine level correlates with glutamate and glutamine in plasma in a murine model [47].

Additionally, mammals have the ability to metabolize large dose of MSG; but, glutamate concentrations in plasma fluctuate during the day and lead to an altered diet, metabolism, and protein turnover [54]. In this context, it has been revealed that the application of lower oral doses could produce altered histomorphological architecture of the brain.

In addition, Walker and Lupien [55] stated that primates, dogs are at lower risk of MSG than the immature blood-brain barrier (BBB) of pups [55]. In contrast, altered plasma glutamate has no such effect on the BBB in adult rodents [11]. However, the nervous structures without BBB like dorsal root ganglia, circumventricular organs, and autonomic ganglia are at risk to acute fluctuations of large magnitude [56]. Body of evidence suggests that an MSG-rich diet alters the cerebrum’s neurochemicals, hippocampus, and cerebellum in adult mammals.

### 3.2 Impact of MSG on the cardiovascular system

Earlier evidence suggested that the administration of MSG in a certain period leads to OS in the heart with elevated levels of cardiac marker enzymes like lactate dehydrogenase (LDH), aspartate transaminase (AST), and alanine transaminase (ALT) [57,58]. In rats with myocardial infarction, MSG induced alteration of the cardiac rhythmic pattern and also tachyarrhythmia [59] in a dose-dependent manner. Researchers performed these experiments that indicate a threat to human health in higher doses (safer limit of MSG used is approximately 16 mg/kg body weight, thereby crossing the boundary has been taken as a higher dose of MSG consumption) and selecting the route of administration respectively. Because the route of administration like intraperitoneal, intravenous and subcutaneous doses are a little bit higher than the human dietary intake [13,43,60], the common metabolic pathways of consumable glutamate cross by these routes [11]. On the other hand, Konrad et al. (2012) proposed that MSG-induced obesity also associates with hypertension, bradycardia, and vagal-sympathetic effects [297]. Moreover, MSG-induced obesity developed by the extra-fats accumulation in adipose tissue due to cholesterol up-regulation ultimately leads to cardiovascular disorders [62]. A significant observation reported by Geha et al. [36] that eating ready-made Chinese foods produced a noticeably rapid heart rate with some other symptoms in humans (e.g., CRS) [36].

From the above reports, it was put forward that MSG-induced cardiac anomaly occurred by altering the cardiac marker enzymes, dyslipidemia, disturbed the balance between free radicals and antioxidants levels, OS, necrosis of cardiomyocytes, cardiac arrhythmia. Still, for better understanding the molecular mechanism of action of MSG-induced cardiac damage and involvement of the proper pathway, further studies are required.

### 3.3 Destructive effects of MSG on the hepatic system

Regular intakes of MSG have been linked with impaired membrane permeability, leads to hepatic fibrosis via distortion of hepatocytes, central vein dilation, lysis of red blood cells, degenerative changes with vacuolation, infiltration of cells in rodents model [11]. However, Takai et al. [63] have reported that MSG causes hyperinsulinemia, hypercholesterolemia, cellular infiltration of eosinophils, lymphocytes, neutrophils, plasmacytes, mast cells, and macrophages, accumulation of liposome in the hepatocytes which finally lead to non-alcoholic steatohepatitis (NASH) in a murine model [63]. Moreover, obesity-induced steatosis and inflammatory response in the liver by MSG lead to hepatocellular carcinoma [64,65]. Additionally, pathogenic activation of Kupffer cells linked with innate effector cells in the damaged hepatocytes and early infiltration of monocytes promote hepatic tissue damage with higher expression of inflammatory M1 macrophages [63,66].

However, human non-alcoholic fatty liver disease (NAFLD) and NASH are caused by the higher MSG consumption in overweight and diabetes patients [67]. On the other hand, Waer and Edress [68] showed that increased MSG intake increases liver glycogen, free fatty acids, phospholipids, and triglycerides content by producing hyperlipidemic conditions. Such elevated glycogen levels in the liver may be due to the high blood sugar and insulin levels [68]. Accumulating evidence suggested that MSG induced OS via disturbing the redox equilibrium by escalating lipid peroxidation with decreasing the endogenous antioxidant levels, which stimulated the generation of collagen fiber in the liver [58,68,69].

Some group of researchers documented that MSG-induced dyslipidemia alters LDL/HDL ratio with the obese condition, increased adiposity without hyperphagic response, hyperinsulinemia by increased insulin level, fibrosis, and steatosis in rodants model [58,70]. Moreover, some studies demonstrated that MSG induced hepatocellular injury via altered liver metabolism via increasing production of aspartate transaminase, alanine transaminase, and gamma-glutamyl transferase followed by inflammation, deterioration of bile ducts, steatosis, infiltration of lymphocytes, monocytes, and macrophages with fibrosis and neoplastic alterations, nodular lesions [11]. Besides, El-Meghawry El-Kenawy et al. [71] also proposed that MSG induced hepatocellular apoptosis via up-regulation of apoptotic mediator proteins such as ki-67and p53 with altered histomorphology of liver, a structural anomaly in mitochondria and endoplasmic reticulum with karyopyknosis which undergoing apoptosis [71]. It is not clear whether there is an association between MSG-enriched foods or diets and hepatocellular toxicity. Therefore, further studies are required to regularly explore the impact of dietary MSG equivalent to human intake as part of an ingredient in ready-made foods on the hepatic system.

### 3.4 Destructive effects of MSG on the reproductive system

On fertility and development of the fetus, logical safety issues arise by preclinical studies on the impact of flavor-enhancing chemicals (i.e., MSG) on chronic exposure to animal models. Previously, researchers showed that MSG altered the typical histomorphological architecture of the testis are associated with various abnormalities in sperm [72]. On the other hand, MSG is also positively linked with altered oocytes and fallopian tubes in rodent models [73,74]. A recent study by Mondal et al. [​^75^​] demonstrated that the consumption of MSG in daily diet has a higher chance of impaired spermatogenesis with higher pachynema in primary spermatocytes and increased primary follicles [75]. Moreover, few other studies documented the harmful quantity in MSG effects on a female reproductive system by ovarian disturbances such as impaired vacuolation of stroma cells, and basement membrane increased in cell size of theca follicular, endosalpinx detached from mesosalpinx [73,74].

In males, the primary sex organ is the most sensitive in the body, more prone to be affected by hazardous environmental factors [76]. Xenobiotic compounds like MS are linked with reduced testosterone [77], altered testicular architecture, testicular hemorrhage, oligospermia with a higher risk of male infertility [78,79]. A recent investigation by Rahimi et al. [9] proposed that MSG induced apoptosis of germ cells in male primary sex organs is responsible for DNA damage via incorporation of labeled 2′-deoxyuridine, 5′-triphosphate (dUTP) at sites of fragmented DNA in a rat model, but the apoptosis was higher in primary spermatocytes than spermatogonia [9]. Earlier reports showed that subtype of metabotropic glutamates (mGlu) receptors such as mGlu1 and mGlu5 are present in testes of animals, humans, and sperm of humans [78,79]. Further activation of cellular development, stability, and distinction-related processes activate calcium (Ca^2+^) waves into the cells via mGlu5 activation since higher amounts of glutamate from the MSG further stimulate glutamate receptors. On the contrary, higher intracellular Ca^2+^ further enters and activates different cellular organelles (e.g., nucleus, mitochondria, endoplasmic reticulum), proteases, and caspases lead to activation of the apoptotic pathway by the consumption of MSG [9].

During pregnancy, MSG consumption induces obesity with downregulation of growth hormones, insulin-like growth factor-1 (IGF-1), reduces IntelliCage place learning and cue discrimination, impairs seizure threshold [80,81]. Moreover, uterine exposure to MSG also altered cerebral morphology and functions by up-regulation of pro-apoptotic genes and thereby genotoxicity in infants [81]. Therefore, from the above evidence, it can reveal that MSG exerted diverse effects on both male and female reproductive systems, but further studies are required for the chronic exposure of MSG on the reproductive system and fetus development with the human equivalent dose of MSG.

### 3.5 Harmful effects of MSG on the excretory system

Kidneys are the central organ in the excretory system, removing toxic materials from the body and regulating the balance between fluid and electrolytes’ quantity in the body. Globally chronic kidney disease (CKD) is identified as a salient noncommunicable disorder; additionally, gut microbiota plays a crucial role in protecting human health, but the gut microbiota’s alteration of the gut microbiota is associated with foods and chronic diseases like CKD [82]. However, *Megamonas, Faecalibacterium*, *Blautia* in gut microbiota are decreased by ingestion of MSG mixed diet simultaneously with an increase of *Collinsella* [83]. On the other hand, the last stage of the patients with CKD lacks *Faecalibacterium,* which is also associated with decreased glomerular filtration rate [83,84].

A significant risk factor of CKD is an unhealthy diet mixed with MSG, which led to obstructive nephropathy, including complete or incomplete blockage of kidneys with a reduced level of stone inhibitors (e.g., citrate, magnesium, etc.) followed by alkalinization of urine [82,85]. In the kidney cell, MSG triggers catabolism of glutamate and forms carbon dioxide (CO_2_) and after that bicarbonate (HCO_3_^−^) respectively; therefore, absorption of HCO_3_- in the circulation causes drainage of excess alkali by the kidney and thereby leads to alkalinization of urine which promotes nephrolithiasis with hydronephrosis [85]. Further, it develops OS, which induced the modification of the fibroblasts into myofibroblast [86] and thereby CKD via the activation of heat shock cognate 71 kDa (e.g., Hspa8 or HSC 70; HSC 70 is the primary inducer of OS) due to inactivation of glutathione-S-transferase [[87], [88], [89], [90]]. Accumulating evidence suggests that OS provokes renal toxicity induced by MSG through N-methyl-d-aspartate receptors (NMDAR) via Ca^2+^ [87,91]. Furthermore, an earlier study proposed that upregulation of NR1 and NR2C subunit of NMDAR linked with nephrotoxicity [92]; glycine and glutamate act as agonistically on NMDAR and stimulates them to produce cell death [93]. Moreover, activation of NMDAR for an extended period of time induced altered intracellular Ca^2+^ dynamics, which stimulates different cellular activities such as nitric oxide synthase and protein kinase C activation [94,95]. Therefore, it can further stimulate the production of free radicals (FA), and oxidative degradation of lipids promoted OS and cellular damage [96]. Additionally, cystine-glutamate chloride-dependent antiporter (CGCDA) regulates the nonvesicular glutamate release, but inhibition of CGCDA leads to reduced intracellular glutathione and thereby ferroptosis [[97], [98], [99]].

A study based on proteomic analysis by Sharma et al. [90] identifies that a higher rate of the Krebs cycle is associated with an increased level of glutamate on the consumption of regular MSG rich diet due to a high level of α-ketoglutarate dehydrogenase, which also leads to OS in cells of the kidney [90]. OS in the urinary system occurs due to uncontrolled metabolism of glutamate for an extended period of time which triggers ROS generation and higher production of free radicals with insufficient endogenous antioxidants, which leads to nephrotoxicity and cellular damage in the renal system [88,89,100,101].

Moreover, cytoplasmic phosphoserine phosphatase (PSP) belongs to the hydrolases class of enzymes, which acts on phosphoric monoester bonds to form serine and phosphate from o-phosphoserine. Pyruvate, cysteine, and glycine are produced from the metabolism of serine in which other than pyruvate, the rest two acts as the precursor molecules for the synthesis of glutathione [90]. Moreover, Sharma et al. [​^90^​] also report that MSG-induced negative regulation of PSP in the renal system also impairs glutathione synthesis [90]. Furthermore, serine and glycine are the primary activators of the glutamate receptor activation, which gets impaired via the downregulation of the PSP in the kidney [102,103]. Additionally, the glycerophosphate shuttle has been activated by MSG via positive regulation of cytosolic glycerol-3-phosphate dehydrogenase (G3PD), which helps transport cytosolic NADH and produced glycerol-3-phosphate (G3P) in mitochondria. MSG-induced alteration of energy metabolism occurs via glycolysis, lipogenesis, and oxidative phosphorylation by G3P [104]. Therefore, glycolytic phosphoglycerate kinase (PGK) acts as a negative regulator by MSG and reduces the catabolism of glucose. Moreover, PGK also plays an essential role in producing energy in the glycolytic pathway, and adenosine triphosphate (ATP) helps regulate this protein in the cell [90]. It can be speculated that, in the catabolism of tryptophan, the positive regulation of 2-amino-3-carboxymuconate-6-semialdehyde decarboxylase (ACMSD) by MSG might be a contributor to the catabolism of amino acid. ACMSD level was higher in MSG mixed high protein diet as compared to a regular healthy diet [105]. Therefore, a high level of glutamate triggers its catabolism, and it also takes part in MSG-induced overactivation of the average Krebs cycle via the up-regulation of α-ketoglutarate dehydrogenase (KGDH) and succinyl-CoA ligase (SCL) [90,106]. Overactivation of the Krebs cycle alters intracellular redox homeostasis. Excess glutamate also triggers the proton gradient in mitochondria; it results in Krebs cycle mediated overabundance of electron donors, leading to excessive superoxide production within the mitochondria [105]. As KGDH is an inducer of OS, it can also be postulated that the MSG-induced generation of ROS occurs via the escalated catabolism of amino acids and also up-regulation of KGDH [90]. Furthermore, Zhang et al. [107] report that SCL has also been indirectly associated with OS via the production of succinate, which further stimulates the production of hydrogen peroxide and thereby generates ROS in the excretory system [107].

Therefore, it is speculated that α-KGDH plays an essential role in MSG induced renal anomaly by overactivation of NMDAR, inhibition of CGCDA, hyperactivation of Krebs cycle by altering the expression of different proteins, which leads to OS and altered the beneficial gut microbiome that should be more vital points for the researcher in their future studies.

### 3.6 Effects of MSG on the immune system

*in vitro* study has been directly linked with the impact of MSG on the immune system by establishing embryonic cell culture models. There was no significant evidence on replication and nuclear division in MSG-induced chromosomal aberrations and exchange in sister chromatids on human lymphocytes. This report has observed that MSG induces genotoxicity on human peripheral blood lymphocytes at the cellular level [26]. Additionally, a higher concentration of MSG exerted a dose-dependent impact on the viability of B cells. According to Jovic et al. [108], metabotropic glutamate receptor (mGluR)-7 is associated with programmed cell death of naive B and memory cells respectively by glutamate [108]. Additionally, various glutamate receptors have been seen in native and memory lymphocytes. In immune cells, OS and apoptosis are seen by differential expression profiles of glutamate receptors [108]. MSG administration in newborn rats disturbs the balance between anti-inflammatory (AI) and pro-inflammatory (PI) cytokines in serum [109]. MSG increases corticotropic-adrenal response, insulin, triglyceride, and leptin levels in newborn rats in the course of an acute phase of inflammation stress. However, the pro-inflammatory cytokine was hindered with average anti-inflammatory cytokines level. From the above evidence, it can be inferred that MSG induced alteration in neuroendocrine-immune and metabolic functions [110]. Moreover, inflammation and damage in the lining of the small intestine are associated with changes in the absorption of lipid on intraduodenal administration of MSG [111]. The body’s defense mechanisms against some infectious diseases were altered by the administration of MSG after birth [112]. Moreover, MSG also causes a reduction of lymphocytes level in the blood without disturbing the basal phagocytosis of neutrophils [11]. However, the earlier reports have also been well documented that MSG is also linked with higher macrophage generation with their phagocytosis on the neonatal period [113]. Furthermore, MSG-induced proliferation in cells of the thymus by escalating the rate of programmed cell death is also linked with an increasing percentage of OS [11]. According to Pavlović et al. [​^79^​], increased levels of malondialdehyde (MDA), xanthine oxidase (XO), with decreased activity of catalase (CAT) is also associated with a higher rate of programmed cell death [79].

In addition, obstruction of macrophage inhibition leads to impairment of functions of delayed-type hypersensitivity (DTH) effector T cells for the investigation of DTH depression in MSG-fed murine model [114]. Another study on the murine model proposed the association between the reduced activities of natural killer cells with quantitatively lower populations of large granular lymphocytes [115]. However, there is no such evidence in human dietary exposure of MSG on the immune system. Therefore, the present review suggests designing an appropriate experimental setup to uncover human dietary exposure of MSG on immune system-related problems, if any.

### 3.7 Role of MSG in tumor progression

Glutamate receptors have also been found in lymphocytes, thymocytes, and some other non-neuronal cells [11]. Accumulating evidence suggested that MSG caused up-regulation of inflammatory cytokines with downregulation of adiponectin in chronic inflammation [116,117]. Moreover, upregulation of some pro-inflammatory factors like resistin, TNF-α, leptin, and IL-6 via peroxisome proliferator-activated receptor-α (PPAR-α) and PPAR-γ activation has also been observed in MSG-induced inflammation in a murine model [118]. Hernández-Bautista et al. [116] demonstrated that altered metabolic activities were also observed in the MSG-fed mouse model of obesity [116]. Moreover, previous studies well documented that MSG-induced obesity and diabetes are positive contributors towards NAFLD, leading to NASH with inflammation and development of primary tumor to hepatocellular carcinoma [64,65,67]. Additionally, cancer development has been linked with stimulated voltage-gated sodium channels, excitatory glutamate, and non-neuronal excitatory receptors mediated hyper-excitability of the cell [119]. Moreover, generation of ROS in the state of OS and abnormal metabolic formation of fat in the liver is correlated with overweight and syndrome-X, which are also identified during the development of hepatocellular carcinoma [11]. The pharmacokinetics of the drug were altered in MSG-induced obese mice via up-regulation mixed-function oxidases such as NAD(P)H: quinone oxidoreductase one and UDP-glucuronosyltransferases 1A. Furthermore, antioxidant defense mechanism and chemoprotection were impaired by the reduced ability of glutathione S-transferases in an obese model [120,121], which either deactivate carcinogens or produce reactive species with increased reactivity contrasted to the primary compound. A recent report by Beyerle et al. [122] showed that there is an interconnection between colorectal cancer with the activities of glutathione S-transferase, cytochrome P450, UDP-glucuronosyltransferase in human [122]. Miyazaki et al. [​^123^​] showed that NASH-associated human hepatocellular carcinoma was also reflected by the diethylnitrosamine-induced hepatocellular tumor in MSG exposed murine model [123].

However, to investigate the relationship between colorectal carcinoma and MSG-induced obesity, Hata et al. [124] have also identified that MSG-induced obesity is at high risk for the development of azoxymethane-induced development of colon cancer via the up-regulation of IGF-1 receptor with an increased level of insulin, cholesterol and blood sugar respectively [124]. In malignant cells, this modified cellular signaling takes a significant part in the synthesis of ATP, controlled the utilization of energy, and sustain the redox equilibrium via the aerobic glycolysis of glucose in the tumor and glutaminase produced glutamate from glutamine; moreover, the citric acid cycle plays a vital role in the production of α-ketoglutarate from this glutamate [125]. Accumulating evidence proposed that enhancement of the obesity-associated tumorigenesis can happen via inhibiting programmed cell death and insulin-IR-ERK1/2 signaling cascade activation [126,127]. Therefore, we can infer that MSG is a positive promoter of initiation of malignancy and tumorigenesis. But further studies are required to explore the generation of tumor and tumorigenesis in humans by the human equivalent dose of dietary consumption of MSG in the diet with proper target pathway of the mechanism of action.

### 3.8 Other harmful impact associated with MSG

Higher incidence of syndrome X has been positively linked with 1 g higher consumption of MSG in the diet [13]. Besides the above pieces of evidence, MSG also exerts diverse destructive impact (Table 1) on human health, such as Type 2 diabetes, by activating N-methyl-d-aspartate receptor (NMDAR) to disturb the functions of β-cell in the pancreas [128,129], hyperphagia, hyperleptinemia, and dyslipidemia by altered lipid profiles with increased leptin level [130,35,131], sleep-disordered breathing (SDB), increased chances of gastroesophageal reflux disease and mandibular retrognathia [43,132,133], etc. Moreover, MSG also de-activates normal thermogenesis processes in brown adipose tissue and thereby leads to altered regulation of thermogenesis [134]. Additionally, MSG has been directly associated with nociceptive responses such as temporomandibular joint (TMJ) syndrome, musculoskeletal pain [11], etc. Furthermore, MSG also acts as a pseudo-allergic chemical that ultimately leads to Chinese restaurant syndrome and exerts inflammatory responses in the human body [35,46,14].

**Table 1:** Adverse impact of MSG along with target pathways in different human and animal models.

MSG induced anomalies	Target pathway	References
Syndrome X	i. Alteration of insulin signaling ii. Activation of ROS/OS-related pathway followed by alteration of redox equilibrium	i.[35] ii.[11,35,65]
Type 2 diabetes	i. Activation of N-methyl-d-aspartate receptors (NMDARs) signaling and NMDARs mediated activation of NF-kB and NOD-like receptor family ii. Insulin resistance (IR) and hyperinsulinemia iii. High glycolytic flux via activation of Ras signaling iv. Activation of the mitochondria-dependent apoptotic signaling cascade	i. [135,129] ii. [136] iii. [137,136] iv. [129]
Hyperphagia, hyperleptinemia and dyslipidemia	Altered metabolic activities, obesity, IR, increased levels of leptin or incompetent binding of leptin to its receptors and altered lipid profile	[35,130,131]
Sleep-disordered breathing (SDB)	Obstruction in respiratory system and increase movement of gastrointestinal tract also leads to SDB via higher incidence of GERD	[43,132,133]
De-activation of thermogenesis	Reduction of thermogenesis in BAT and thereby decreases the normal thermoregulatory mechanism	[134]
Nociceptive response	Increase interstitial concentration of glutamate in the facial muscle with musculoskeletal disorders followed by nociceptive response	[35]
Allergy, eczema and MSG symptom complex	MSG acts as pseudo allergens to cause allergic responses	[35,46,138,12]

#### 3.8.1 Syndrome X

A collection of diseases including obesity, hyperglycemia, dyslipidemia, high blood pressure, diabetes steatosis of the liver and cardiovascular disorder (CVD) called metabolic syndrome [35]. Recently, some other names have been attached to it, such as insulin resistance syndrome, Reaven syndrome, dysmetabolic syndrome, and syndrome X (SX) [139]. Moreover, SX patients must have at least hypertension, high level of triglycerides, decreased level of ‘good cholesterol’ and elevated level of blood sugar, which were demonstrated by the American Heart Association, International Atherosclerosis Society, World Heart Federation, International Diabetes Federation, International Diabetes Federation, International Association for the Study of Obesity, and National Heart, Lung and Blood Institute; additionally altered insulin signaling in the brain and release of free fatty acids from adipose tissue also triggers glucose production in the liver and very-low-density lipoprotein (VLDL) simultaneously reduced the level of ‘good cholesterol’ [35]. Moreover, some factors including lack of physical activities, inflammatory response for the prolonged period, an elevated level of leptin, malfunction of mitochondria, genetic susceptibility, pancreatic dysfunction with the destruction of β-cells and increased level of free fatty acids, reduced uptake of glucose in skeletal muscle with higher production in the liver stimulated the progression of SX via alteration of insulin signaling [35]. However, MSG-induced generation of ROS altered the balance of antioxidant and free radical balance, and hepatic anomalies are also linked with the metabolic syndrome in the hepatocellular carcinoma model [11,35,65]. Accumulating pieces of evidence suggested that redox equilibrium, adipose tissue metabolism, endocrine glands, innate immunity, endothelial function, fibrinolysis, and coagulation-related anomaly have also been correlated with SX [35,140,141]. Furthermore, epidemiological surveys on humans also proposed that MSG in the diet also promoted SX [13].

#### 3.8.2 Type 2 diabetes

Activation of NMDAR leads to failure of functions of the β-cell in the pancreas by reducing the mass of the β-cell, insulin secretion leads to type-2 diabetes due to the higher intake of glutamate [135]. Insulin resistance (IR) and hyperinsulinemia are responsible for developing obese neonatal rats exposed to MSG [136]. Furthermore, in hypertrophic islets of the pancreas, high glycolytic flux might be via Ras signaling activation [137] with upregulation of GLUT2 linked with hyperglycemia and diabetes [136].

Some other studies well demonstrated the hypertrophic, hyperplastic effects of MSG on islets of the pancreas with reduction of α-cells, *δ*-cells, and acinar cells [142]. Furthermore, a recent study by Boonnate et al. [​^142^​] proposed that MSG induced diabetes by reduction of mass of β-cell of the pancreas with higher production of 4-hydroxy-2-nonenal in islets of the pancreas via oxidative stress [142]. However, fibrosis and β-cell destruction of the pancreas occurred by an increased level of extracellular glutamate [143]; it further diminished the cysteine precursor for the synthesis of glutathione by impeding the counter-transporter of glutamate-cysteine in β-cell of the pancreas and finally contributed to oxidative stress-mediated cell death [142]. Furthermore, excess glutamate in the body stimulated high glucose-induced overactivation of glutamatergic N-methyl-d-aspartate receptors (NMDARs), signaling mediated activation of NF-kB and NOD-like receptor family member pyrin domain-containing protein 3 (NLRP3) inflammasomes which diminished the function of β-cells in the pancreas by downregulation of transcription factors [128,129]. Moreover, it may also cause mitochondrial dysfunction, leading to programmed cell death via activation of the mitochondria-dependent apoptotic signaling cascade by up-regulation of pro-apoptotic and downregulation of anti-apoptotic protein [129]. Therefore, all evidence suggested that MSG induced chronic pancreatitis, failure of function of β-cells in the pancreas to promote diabetes, and thereby programmed cell death.

#### 3.8.3 Hyperphagia, hyperleptinemia, and dyslipidemia

Hyperphagia is an inherited condition in which a person has an extreme desire to eat food without satisfaction [144]. Adipocytes released leptin that binds with the receptor situated in the arcuate nucleus of the hypothalamus, which incited such responses [145]. On the contrary, Afifi and Abbas [130] proposed that MSG induced hyperphagia by an increased leptin level in serum because of the incompetent binding of leptin to its receptors [130]. High serum leptin is also linked with the body mass index (BMI) and higher adiposity [146]. Moreover, MSG also altered metabolic activities related to hyperphagia with obesity which is also correlated with IR, increased levels of leptin (i.e., hyperleptinemia), and the altered ratio of bad cholesterol/good cholesterol (i.e., dyslipidemia) [35,131].

#### 3.8.4 Sleep-disordered breathing (SDB)

Regular consumption of MSG mixed foods is linked with partial or complete cessation of breathing and obstructive sleep apnea syndrome (OSAS), a global public health issue. Moreover, it could be a positive contributor to pulmonary disorders, cardiovascular disorders like morbid conditions. It occurred due to the obstruction of the pharynx or breathing pause or insufficient ventilation during the sleeping period and produced snoring without disturbing the sleep and depended upon an individual’s age and body weight. Furthermore, escalated movement of the gastrointestinal tract by MSG leads to the higher incidence of gastroesophageal reflux disease (GERD), which also linked with OSA, are more common among Asians, according to their cranial architectural aspects and a higher rate of mandibular retrognathia [43,132,133].

#### 3.8.5 De-activation of thermogenesis

Thermogenesis is a process of the generation of heat in animals or the human body. Very little information is available - although an earlier study proposed that MSG promoted significant loss of body temperature without mobilized brown adipose tissue (BAT) when exposed to the cold environment (e.g., 4 °C), depended on the exposure time in the murine model. They suggested that MSG blunted the mechanism of activation of thermogenesis in BAT and diminished the regular thermoregulatory tool for thermogenesis [134].

#### 3.8.6 Nociceptive response

MSG exerts an adverse impact on human beings by escalating the interstitial concentration of glutamate in the facial muscle of mastication in patients with temporomandibular disorders and triggered the severity of pain sensation [35]. It has also been observed from a recent study by Vellisca and Latorre (2013) that a typical musculoskeletal pain was intensified by MSG in-patient with fibrositis [298]. On the contrary, a recent study proposed that MSG is directly linked with a high concentration of nitrates in the brain by decreasing the thermal nociceptive threshold [147]. Therefore, it can be proposed that there could be a possible link between intakes of MSG in ready-made foods with the nociceptive stimulus to intensify the pain sensation, and further studies are required to evaluate the long-term administration of the minimal dose of MSG on the nociceptive threshold.

#### 3.8.7 Allergy, eczema, and MSG symptom complex

MSG-enriched foods cause burning and unusual pain sensation in the chest, emotional distress, migraines, atypical facial pain or pressure, stiffness of muscle, involuntary and temporary reddening of the skin, pins-and-needles sensation [42,44,148]. However, after consumption of ready-made foods mixed with MSG caused skin rashes, inflammation on the skin followed by reddening and thereby cracked skin, asthma, dysfunction of peripheral nerves (e.g., numbness, weakness), ventricular arrhythmia, abdominal irritations, and also CRS [12].

Additionally, eczema with chronic fluctuation of body temperature is a condition which supposed to be caused by consumption of MSG and other food additives-enriched diet; on the contrary, reduction of the use of food additives as pseudo allergens like MSG in the diet decreased the chance of such allergic reaction in the skin [35]. Furthermore, MSG acts as a pro-inflammatory mediator in the immune-linked pathogenesis of eczema [149,150]. Therefore, intake of MSG in foods or diet directly correlated with atopic dermatitis or eczema-like skin infections.

In addition, on the harmony of allergy and skin infection by the exposure of MSG mixed ready-made foods or junk food, Chinese restaurant syndrome (CRS) is also known as MSG symptom complex (MSC); precisely, a group of neurological syndromes similar to the MSG-induced allergic responses [46,138] arises just after twenty minutes of taking MSG mixed diet. But it did not occur in all kinds of people; it depends upon the MSG-sensitive person [62]. A recent report by Bawaskar et al. [24] proposed that without healthy-solid-diet with good nutritive value, higher consumption of MSG mixed foods with low nutritional value are directly associated with increased risk for the incidence of MSC [24].

## 4 Effects induced by HLD, as a central component of the modern diet, on human homeostasis

In today’s world, people often prefer a high lipid diet daily with very minimal physical activities. This has led to epidemically obese conditions and obesity-linked metabolic diseases [3]. Further, it can develop an imbalance of energy homeostasis and cause a burden on global diseases like dyslipidemia, hyperlipidemia, hyperglycemia, diabetes, liver-related problems, cardiovascular problems, OS, and cancer. However, not all the high lipid die ingredients are toxic; mainly the saturated fatty acids, trans-fatty acids, and hydrogenated fats are the main ingredients of an un-hygienic diet [3,151]. Besides the use of MSG, hydrogenated palm oil in vanaspati also acts as a flavor enhancer in different HLD [62]. Moreover, earlier studies in adults have shown that saturated fatty acids, trans-fatty acids containing diets are positively associated with increased low-density lipoprotein (LDL), total cholesterol (TC) mediated cardiovascular disorder [3], coronary heart disease, and increased the mortality rate [152]. Generally, coronary heart disease and cardiovascular disorders are observed in older age; dyslipidemia and other cardiovascular diseases in older people are directly linked with the previous atherosclerotic lesions in coronary arteries and aorta in childhood [153]. In childhood, increased TC and LDL levels are also linked with increased risk of cardiovascular disorder in later life with subclinical atherosclerosis by condensing the tunica intima and tunica media layer of the carotid artery [153]. Hence, it can be predicted that high intake of dietary saturated fatty acids and trans-fatty acids are positively associated with TC and LDL levels in adults and thereby cardiovascular disorder.

A cohort study of Bendsen et al. [​^152^​] demonstrated that high consumption of trans-fatty acid is also linked with an increased chance of coronary heart disease and morbidity [152]. Diets containing low carbohydrates but high amounts of saturated fatty acids, hydrogenated fats, and trans-fatty acids may increase the risk of cardiovascular disorders [3,154] as there is a positive correlation between saturated fatty acids and bad cholesterol (low-density lipoprotein) with increased risk of vascular malfunctions [153,155]. Due to differential effects of saturated fatty acids on the concentration of the subclasses of LDL, some meta-analyses and systematic reviews have reported that saturated fatty acids are not causal agents for cardiovascular disorders; moreover, it has also been reported that cardiovascular diseases are more likely to be linked with small and medium LDL particles than the larger one [156]. Dreon et al. [​^157^​] well documented that higher consumption of palmitic acids and myristic acids like saturated fatty acids positively associated with increased levels of larger LDL particles in plasma without alteration of the concentration of apolipoprotein B (apoB) concentration [157]. Also, a higher concentration of low-density lipoprotein-cholesterol (LDL-C) is positively linked with increased concentrations of cholesterol-enriched larger LDL, but no changes could be found in the concentration of apoB due to the exchange of saturated fatty acids for monounsaturated fatty acids in the diet, in the context of low levels of the intake of carbohydrate-rich diet [158]. Increased HDL-cholesterol (HDL-C) has also been positively associated with saturated fatty acids in the diet, which can balance the atherogenic effect of elevated LDL-C [3,156].

One of the most common dyslipidemia is atherogenic dyslipidemia with increased plasma triglycerides, reduced HDL-C, and elevated levels of small, dense LDL particles; atherogenic dyslipidemia is also associated with IR and obesity [158]. The expression of LDL is dependent upon two phenotypes; phenotype A is the individuals with predominantly larger particles of LDL, and phenotype B is characterized by the individuals with an abundance of small, dense particles of LDL [156]. Bodyweight, genetic liability, uptake of dietary micronutrient influence the LDL phenotype B expression but higher uptake of carbohydrate stimulates elevated levels of very-low-density lipoproteins (VLDL) that promotes the acute increase or transformation of small, dense LDL particles and phenotype B [159]. HLD has also been associated with disturbance in the microbiome of the gut with increased permeability of gut and inflammation also [99,160]. Moreover, in synchrony with earlier studies, it can be proposed that saturated fatty acids have diverse effects on atherogenic LDL particles that depend upon the amount of consumed saturated fatty acid in the context of diet, mainly with the consumption of carbohydrate, liability to atherogenic dyslipidemia.

**Fig. 3:**
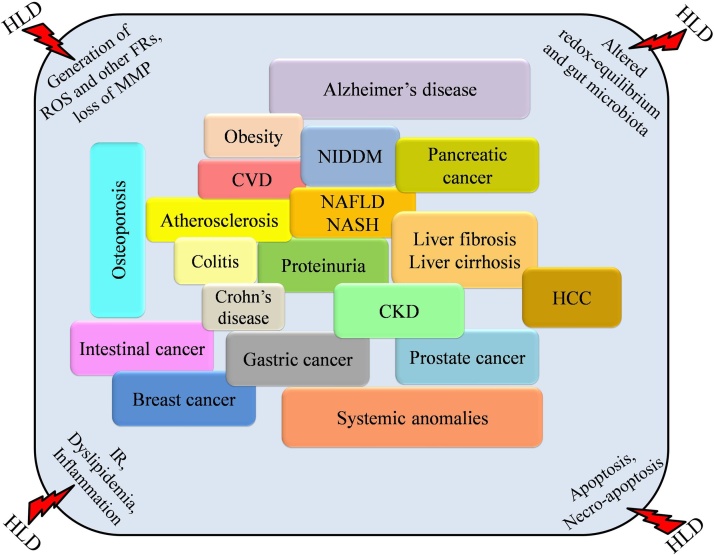
HLD induced altered homeostasis and metabolic disorder leads to systemic anomalies.

To reduce the global burden of diet-related metabolic disorders, we should understand the lipid-enriched diet’s pathogenesis, which induces systemic damage. The present review has been trying to pinpoint various causes and effects of diseases induced by such high lipids-based diets. We have also discussed the viable strategy of managing such diet-activated conditions. Fig. 3 shows the overall deleterious impact of HLD.

### 4.1 Harmful impact of HLD on the central nervous system

Previously, researchers documented the positive link between HLD and central nervous system disorders by an inflammatory response in the brain and spinal cord to develop CNS-related disorders [161]. Kothari et al. [​^162^​] reported that lipid-enriched diets promote alteration in the brain’s insulin signaling and impaired mental activities in a murine model [162]. Accumulating evidence demonstrates that lipid-enriched diets delay memory, increase anxiety and lead to over-generalized behaviors by altering the gut microbiota, while their long term consumption further promotes amyloid-peptide deposition, linked with Alzheimer's disease, in the brain of a murine model [163,164]. Besides, lipid-enriched diets are also positively correlated with OS by the generation of ROS, peroxynitrite, and superoxide in the brain, which exhibit transparent declination in cognition [161]. Therefore, it can be inferred from the accumulating evidence that lipid-enriched diets are positively linked with OS in the brain with impaired cognitive functions and cerebral deposition of amyloid β-peptide, which prompts Alzheimer’s disease [161,165]. Besides, lipid-enriched diet-induced OS impeded insulin signaling by up-regulating the GSK-3β and hyperphosphorylation of protein with increased amyloid β-peptide, promoting neurodegeneration and reduced synaptic plasticity; these factors ultimately lead to the occurrence of Alzheimer’s disease [162]. It was also well documented by Petrov et al. [​^166^​] that HLD disrupted the BBB with limited transportation of glucose and insulin to the central nervous system, which provoked hyperinsulinemia to some extent can primarily stimulate deficiency of insulin in the brain, which down-regulates the expression of insulin-degrading enzyme and less accumulation of amyloid β-peptide [166]. Moreover, abnormal behavior like conditions such as depression, anxiety may be developed by HLD via increase expression of pro-inflammatory IL-1β in the amygdala and alteration of GABAergic neurotransmission in the dorsomedial part of the hypothalamus [161].

So, it is now clear that HLD induced brain damage by some potential mediators such as IR, alteration of integrity of BBB, inflammatory responses, and generation of ROS may ultimately disrupt the brain’s normal functioning and gradually develop neurodegenerative disorders like Alzheimer’s disease.

### 4.2 Association of HLD with an inflammatory response in different parts of the body

A recent study by Guillemot-Legris et al. [​^167^​] has proposed that intake of lipid-enriched diets produced some inflammatory responses in the CNS, hypothalamus, adipose tissue, liver, intestine, and skeletal muscle [167]. Primarily, lipid-enriched diet stimulates the gut microbiota changes with immediate impact on intestinal free fatty acids (FFAs), which leads to chronic systemic inflammation followed by the release of immune-related cells derived pro-inflammatory cytokines with activation of the innate immune system [161]. An earlier study suggests a positive correlation between the altered gut microbiota in adiposity and inflammation [168]. Additionally, some researchers have also proposed that people with plenty of bacteria are associated with higher levels of inflammation with morbid overweight than the scarcity of bacteria in a person [169]. So, inflammatory response and reduction of the diversity of bacteria, specifically *Bacteroidetes*, with relatively high populations of *Firmicutes* are found due to intake of HLD in both animals as well as humans.

Toll-like receptor (TLR) signaling pathway has been activated by the rearrangements of gut microbiota, which further reduces the intestinal potential and simultaneously enhances the permeability of the intestine; thus, lipopolysaccharides enter into the intestine as endotoxins and transfer of such endotoxins into the systemic circulation [161]. However, it was also evidenced that a higher quantity of FFAs in the lipid-enriched diets explicitly worked on the cells of the intestine; furthermore, higher productions of IL-1β, IL-6, TNF-α in like an inflammatory mediator in the gut are directly connected with higher quantities of the release of lipopolysaccharides or FFAs [61,161]. Few earlier studies also documented that there is another step of high lipid diet-induced inflammation where lipopolysaccharide are concerned with systemic low-grade inflammatory response by enhancingthe the production of lipopolysaccharides in the intestine with inflammatory mediators and FFAs into the circulation (i.e., systemic and portal circulation) [61,170]. In addition, macrophages in the circulation showed increased TLRs expression when large amounts of lipopolysaccharides and FFAs were present in the plasma, which further facilitated to activation of the macrophages (M1 phenotype) to produce inflammatory mediators [161]. Such inflammatory mediators stimulated the pathways related to inflammation of the brain before the development of obesity. Activation of hypothalamic IkB kinase b (IKKb)/NF-kB signaling pathway occurred by higher production of cytokines and FFAs. There are two main ways of such path either via the activation of cell surface TLR or by activation of different stress-related pathways at the cellular level (e.g., endoplasmic reticulum or OS) in the hypothalamus [[171], [172], [173]]. Therefore, reduced levels of main adipokines (e.g., leptin) and insulin sensitivity initiate inflammatory gene expression in the hypothalamus by activating IKKb/NF-kB signaling [161,171]. Simultaneously, peripheral inflammation occurred via the activation of macrophages related to plasma inflammation, which can spread over the blood vessels, adipose tissues, pancreas, muscular tissues, etc. [161,174]. In the fatty tissue, accumulation of M1 macrophages occurred via the CD8^+^ T cells accumulation [175]. We can speculate that these macrophages play a pivotal role in developing chronic inflammation-linked metabolic anomalies [176].

A report by Lumeng and Saltiel [​^174^​] stated that adipose tissue declines to store additional lipids during HLD induced stress condition; on the contrary, these excess lipids are deposited in some other areas of the body such as blood vessels, liver, skeletal muscle, and pancreas [174]. Moreover, systemic inflammation provoked by such abnormal accumulation of excess lipids stimulate the up-regulation of some pro-inflammatory and M1 macrophages enrolment [161]. Additionally, the gastrointestinal tract derived lipopolysaccharides, FFAs, and some other inflammatory mediators (e.g., pro-inflammatory cytokines) are quantitatively higher in the liver [61]; which further promote systemic as well as hepatocellular inflammation via the Kupffer cell actuation and natural killer T cells accumulation in the liver [177].

Now, it can be conjectured that there is a synergistic effect of lipid-enriched diet on complex signaling interconnection of various organs of the body with low-grade systemic inflammatory responses; because of such inflammation, fat cells failed to eliminate FFAs and leads to the development of noninsulin-dependent diabetes mellitus (NIDDM), hepatic disorders, cardiovascular disorders (CVD), atherosclerosis and some types of malignancy.

### 4.3 Role of HLD in development of IR and NIDDM

IR is a condition where the body fails to acknowledge circulating insulin with reduced clearance of glucose from a system with a reservoir of glucose in different tissues such as adipose tissue, liver, and muscle and finally developed IR incidence [178]. Lipid-enriched diets induce IR and thereby alter the normal functions of the β cell of the pancreas. Short-term administration of HLD leads to the systemic failure of response to circulating insulin and leads to the progression of hyperglycemia in murine models [179]. Accumulating evidence suggested that long-term administration of lipid-enriched diets in murine models showed higher levels of intolerance of glucose, reduced sensitivity of insulin, IR, beginning of compensation of pancreatic β cells [180,181]. However, some recent studies reported that HLD induced inflammatory responses and the developments of IR are interconnected [182,183]. Inflammatory responses in the hypothalamus by consumption of lipid-enriched diet hindered the secretion of insulin from pancreatic β cells and activity of insulin on the periphery; macrophages assembled in the islets of the pancreas and inhibited the normal function of β cells when pancreatic β cells produced pro-inflammatory cytokines [161]. Moreover, higher generation of 12-hydroxyeicosatetraenoic acid (12-HETE) occurred by the activation of the activity of 12-lipoxygenase in pancreatic β cells via the FFAs, cytokines, glucose intolerance by the consumption of HLD; this 12-HETE also an inducer of OS, which also inhibited the function of the nuclear factor erythroid 2-related factor 2 (Nrf2), ultimately leads to glucose intolerance and programmed cell death of β cells of the pancreas [184]. Hence, lipid-enriched diets stimulate the release of insulin from the pancreatic β-cells lead to hyperinsulinemia.

We have already discussed the concept of the “two-hit” hypothesis where the first hit is concerned with the gathering of fatty acids or triglycerides in the hepatic tissue and ultimately give rise to IR, and the second one is linked with inflammation and OS, which resulted in hepatic damage. But currently "multiple-hit model" has been popularized instead of the old “two-hit” model to demonstrate systemic anomalies, which is based on the connection between genetic and environmental factors to produce metabolic dysfunctions and multiple organ damage [185]. Moreover, adipose tissue lipolysis occurred due to the dual role of inflammation. Hyperinsulinemia permits glycerol and FFAs to circulate to the hepatic tissue and further promote gluconeogenesis; glycolysis in muscle is enhanced by the high concentration of insulin and production of lactate. This further moves on to the liver and is utilized as a substrate for gluconeogenesis in hepatic tissue; eventually, a higher rate of gluconeogenesis produces large amounts of glucose in the hepatocytes and developed systemic IR [161].

Subsequently, systemic IR with reduced secretion of insulin from the β cells of the pancreas ultimately leads to NIDDM [178]. Additionally, HLD induced obesity-linked pancreatic anomaly is controlled by a G-protein coupled adenosine A_2B_ receptor found in insulin-positive β cells of islets of Langerhans, which is also an accepted promoter of inflammation [186]. Furthermore, it has also been reported in an earlier review study that, specifically, macrophage G-protein coupled adenosine A_2B_ receptor acts as a promoter of HLD induced NIDDM [161]. It may be a viable therapeutic strategy of management against NIDDM and regulate the macrophage G-protein coupled adenosine A_2B_ receptor by manipulating the monocytes either genetically or pharmacologically. In this context, it was clearly stated that the development of IR and NIDDM are linked with each other in association with large amounts of high lipid diets.

### 4.4 Impact of HLD on the hepatic and cardiovascular system

Saturated fatty acids and hydrogenated fats in HLD alter the redox homeostasis, which is further provoked by pro-inflammatory cytokines [3]. Moreover, inflammatory mediators like IL-6 and TNF-α plays an essential role in the development of NAFLD in human and key stimulus for production of various proteins in the liver (e.g., fibrinogen, high-sensitivity C-reactive protein) in acute-phase of response; where a higher level of high-sensitivity C-reactive protein (hs-CRP) indicates an increased risk of cardiovascular disorder (CVD) in a patient with NAFLD mediated NASH [187]. However, HLD also exerts deleterious effects on cardiac tissue architecture by reducing the number of cardiac muscle fiber, inflammatory responses, deposition of lipids within the myocardium, dissolution of the nucleus, which indicate muscle filaments degeneration and necrosis of cardiac muscle, respectively; in hepatic tissue with cytoplasmic vacuolization, accumulation of lipids, infiltration of inflammatory cells, necrotic cells and cellular lysis respectively [3].

In addition, saturated fatty acids, trans-fatty acids, hydrogenated fat in ready-made foods altering the LDL/HDL ratio, which leads to dyslipidemic conditions. HLD also alters some liver and heart functional enzymes and stimulates hepatic and cardiac injury, respectively. Palmitic acid, lauric acid, myristic acid, and hydrogenated fats in HLD increase the SubG1 phase by arresting the G0/G1 phase, loss of mitochondrial potential, and decreasing viability of hepatocytes and cardiomyocytes, respectively, which further indicates programmed cell death of hepatocytes and cardiomyocytes. According to the study of Banerjee et al. (2020), HLD induces NAFLD mediated hepatic and cardiac as well as systemic damage [3]. But, the NAFLD is a complex disease that is associated with various factors, and to explain this disease “multiple-hit model” is appropriate instead of the “two-hit” hypothesis [185]. In patients with NAFLD, a multi-functional gene liver X receptors-α (LXRα) is linked with the accumulation of fats in hepatocytes with inflammatory responses and hepatic fibrosis [188]. According to Yasutake et al. [​^189^​], cholesterol intake is dramatically increased in non-obese NAFLD patients than obese NAFLD patients [189]. High cholesterol significantly stimulates the progression of steatosis in the liver. Accumulation of cholesterol in the liver leads to activation of liver X receptor α-sterol regulatory element-binding protein-1c (LXRα-SREBP-1c) pathway, which is the critical signaling cascade for the development of NAFLD [188].

Song et al. [​^188^​] proposed that HLD induces hyperlipidemic effects and metabolic disturbances by increasing the visceral fats, inflammatory factors, OS, and IR. Anti-inflammatory adiponectin acts as a mediator of IR and inflammatory response, promoting the development of NASH [188]. Furthermore, different isoforms of transforming growth factor-β (TGF-β) (e.g., TGF-β1, TGF-β2, and TGF-β3) promotes necroinflammation, development of hepatic fibrosis and steatohepatitis through Kupffer cells, stellate cells, and endothelial cells of the liver [190,191]. Moreover, HLD alters the lipid metabolism, energy balance, and inflammatory response at the genetic level by transcription factors such as PPAR-α and PPAR-γ. Some earlier studies well documented that PPAR-α and PPAR-γ regulates mitochondrial as well as peroxisomal β-oxidation and Ω-oxidation (microsomal). Still, HLD down-regulated the expression of PPAR-α and PPAR-γ, which altered the β-oxidation and Ω-oxidation regulatory mechanism [188]. Souza-Mello [192] suggested that inactivation of the transcription factors (i.e., PPAR-α and PPAR-γ) also promoted reduced expression of anti-inflammatory adiponectin and increased IR. Still, activation has a beneficial impact [192]. Hence, it can be suggested that HLD with a high amount of saturated fatty acids (e.g., palmitic acid, lauric acid, myristic acid), trans-fatty acids, hydrogenated fats activated LXRα-SREBP-1c pathway associated NAFLD, dyslipidemia, altered hepatic and cardiac metabolism, increased expression of certain pro-inflammatory factors, generation of ROS, a decrease of mitochondrial membrane potential (MMP) and necro-apoptosis in hepatocytes and cardiomyocytes respectively.

However, an earlier study demonstrated that 20 % of the fat-containing diet increased nitric oxide production (NO) and thereby OS, resulting in larger lesion size after myocardial infarction in a rodent model [193]. Effector T cells mediated infiltration of fatty tissuesand vascular tissues are linked with some cardiovascular problems in lipid-enriched diet-induced inadequate systemic inflammatory response; an earlier cohort study demonstrated the significant difference between the obese patient and lean persons on the pro-inflammatory effector memory-like phenotype (e.g., C-X-C motif chemokine receptor 3^+^) where the obese patients have a higher number of C-X-C motif chemokine receptor 3^+^ (CXCR3^+^) effector memory T cells. This phenotype was also found in murine models. Moreover, it was also well documented that the PI3K p110d-Akt signaling pathway is responsible for the differentiation of CXCR3^+^ effector memory T cells [194]. Additionally, malfunctioning of vascular endothelium due to regular consumption of lipid-enriched diet plays an essential role in the incidence of cardiovascular disorders [82,195]. Martins et al. [196​] reported that in erythrocytes, the l-arginine-NO pathway is halted by the feeding of animal fat-enriched HLD (e.g., fat from pig) in the murine model [196]. NO maintains the vascular functions by concealing the aggregation of platelets and promoting NO pool within the vascular system; moreover, there is an inhibition of vascular function because of reduced bioavailability of NO, which increases cardiovascular disorders. So it may be hypothesized that IR, free fatty acids (FFAs), and higher blood sugar levels autonomously alter the endothelial homeostasis by intake of HLD and progression of ill cardiovascular effects [161,197]. Small cytokines, signaling protein molecules on binding with erythrocytes through Fy glycoprotein or cluster of differentiation-234 protein causes up-regulation of such chemokines in 60 % lipid-enriched diet-fed murine model. Furthermore, there was a high level of cholesterol and externalization of phosphatidylserine from the membrane in erythrocytes of lipid-enriched diet administered mice associated with a higher rate of phagocytosis of erythrocytes by macrophages outside and by spleen inside which, further promotes atherosclerosis [197]. Here it can be suggested that HLD is a contributor to cardiovascular disorders via adipose tissue and vascular tissue infiltration via effector T cells, malfunctions of the endothelium, and erythrocytes.

### 4.5 Harmful effects of HLD on the digestive system

Lipid-enriched diets are major contributors to inflammatory responses in the intestine and adjacent tissue dysfunction. The consumption of HLD can intensify chemical-induced colitis via the up-regulation of inflammatory cytokines; moreover, in Mdr1a^−^/^−^ murine model, ileitis can also aggravate tissue damage in the mucosal area and instinctive colitis in Muc2^−^/^−^, TNF^ΔARE^ murine model [161].

Previously, some researchers suggested that intake of lipid-enriched diets have also been positively correlated with higher-risk inflammatory bowel disorders (IBDs) with chronic inflammation in the digestive tract and other functional bowel disorders in human [198,199]. Moreover, high consumption of dietary lipid is positively connected with a higher risk of Crohn’s disease-like inflammatory bowel disorders due to stimulation of an inflammatory response in the gut and disturbance of microbiome linked with alterations of mucosal immunity in the colon [161]. Additionally, a fat-enriched diet causes an imbalance of protective bacteria (e.g., *Bacteroidetes*) and harmful bacteria (e.g., *Proteobacteria*, *Firmicutes*) permeability of the intestine, maladaptation mucosal bacteria with modifying the composition of the normal microbiota. G protein-coupled receptor 43 (GPR43) and peroxisome proliferator-activated receptor-γ (PPAR-γ) were reduced by HLD, which further stimulate these effects. Hence, activation of GPR43 and PPAR-γ signaling pathways could be taken as a viable approach of management for curing the patient with inflammatory bowel disorders (e.g., Crohn’s disease) [200,201]. Additionally, anomalies in gastrointestinal movements have also been concerned with HLD induced obese mice activated inflammatory response and dysfunction of the intestine with altered morphology of the intestinal tract’s intrinsic nervous system. The mechanism behind the intestinal macrophages mediated abnormal movements of the colon that occurred in the HLD induced an obese model is via G-protein coupled adenosine A_2B_ receptor-directed excitatory tachykinergic pathways [161].

### 4.6 Role of HLD in urinary system related problems

We have already mentioned that the role of gut microbiota protects the health of humans. In keeping with the effects of MSG, regular as well as prolonged intake of HLD reducing the population of *Bacteroidetes* and enhancing the abundance of both *Firmicutes* and *Proteobacteria* in the intestine leads to OS mediated kidney damage [82]. A cross-sectional analysis demonstrated a robust connection between the consumption of HLD enriched with saturated fatty acids and proteinuria, which is linked with damage of normal functions of kidneys which is more significant than 19,000 adults above the age of 45 years [202]. To divulge the possible mechanism for the progression of malfunction of the excretory system or the primary excretory organ through the consumption of lipid-enriched diets, researchers are now increasingly using rodent models for further investigations with proper maintenance of biodiversity. Ebenezer et al. [203] reported that administration of lipid-enriched diet produced hyperalbuminemia via the up-regulation of TNF-α, NF-kB like inflammatory factors, and nicotinamide adenine dinucleotide phosphate (NADPH) oxidase in the outer part of the kidney, which is a marker of OS [203]. A similar type of metabolic and molecular impact of lipid-enriched diet was reported in an earlier study by using the Sprague-Dawley rat model [204] with a short duration of (i.e., 42 days) experiment as compared to the previous (i.e., 70 days) one [203]. In addition, an ApoE^−/−^ the murine model has been used to develop hyperlipidemia induced inflammatory responses on the progression of renal malfunction by the administration of HLD [161]; which further showed a significant up-regulation of pro-inflammatory factors like IL-6, TNF-α followed by the development of inflammation in kidney, the proliferation of mesangial cells and hyperalbuminemia. Moreover, the anti-IL-6 receptor antibody acts as a protective agent against such HLD induced inflammation-mediated anomalies in the kidney in the murine model. Furthermore, it was also documented that renal sterol-regulatory element-binding protein (SREBP1) is upregulated by the action of pro-fibrotic growth factors and pro-inflammatory cytokines. It has been proposed that up-regulation of SREBP 1/2 is connected with overweight, increased blood sugar, and increased insulin by the administration of 60 % of fat in the diet in a murine model [205]. Another study by Wang et al. [​^206^​] demonstrated that inflammation in the kidney and hyperalbuminemia could be inhibited by the knockout of SREBP1 in the murine model [206]. Hence, it can be postulated that SREBP1 intensifies the kidney’s inflammatory response, which further leads to HLD induced CKD.

### 4.7 Deleterious impact of HLD on the skeletal system

A mesenchymal stem cell is an origin of lipocytes, fat cells, and osteoblast, which further leads to the interactivity between formations of lipocytes, fat cells with the growth of osteoclasts that can be influenced by the consumption of a high amount of lipids in the diet [161].

Body of evidence on the incidence, distribution, and control of diseases well documented that high dietary lipids are directly correlated with osteopenia and increased chance of osteoporosis [[207], [208], [209]]. Moreover, 21.1 % of lipid-enriched diet-induced osteopenia with OS in a murine model [210]. Similarly, high lipid diets are also associated with a reduction of the number of trabeculae and volume of trabecular bone in the shinbone [211], which produced a harmful impact on the shape and number of the bone. On the contrary, some studies on animal and human model reported that higher physical stress is concerned with a higher intake of dietary lipids induced increase of bone mineral density [212,213] and higher bone mass for the short-term intake whereas the long-term intake obesity-induced showed negative impact [214]. Hence, it is now clear that dietary intake of a high amount of lipids can primarily build up bone mass for physical requirements, but dysregulation of metabolism gradually weakens the process of ossification [161].

Another experimental study on a murine model suggested that lipid-enriched diet also positively correlated with increasing adipocytes in bone marrow after injection of B16F10 melanoma cells in normal mice, which causes alteration of microenvironments of hematopoietic stem cells in the bone marrow and create a microenvironment for malignant cells followed by up-regulation of their growth by Janus kinase 2 (JAK2) and IL-6. Furthermore, malignant cells in the bone marrow are directly communicated with fat cells and augmented large multinucleated bone cells in the bone marrow via the expression of bone sialoprotein I (BSP1). In the context of lipid-enriched diet-induced obesity, the IL-6-JAK2-BSP1 signaling pathway plays a significant role in contributing to the microenvironment for metabolic tumors [215]. However, due to the high intake of fatty foods, the hormone leptin plays a vital role in regulating bone deposition by high lipid diet [216].

A large amount of lipid-based diet controls bone growth and resorption, leading to bone fractureobesity-induced (i.e., osteoporosis) via the IL-6 and leptin.

### 4.8 Different types of malignancy caused by HLD

Lipid-enriched diets are also associated with certain types of malignancy in humans as well as animal models via inflammatory response, metabolic alteration, and affecting some signaling cascades. High lipid diet-induced cancer in liver, breast, stomach, intestine, pancreas, and prostate in different human and animal models via targeting different cellular signaling pathways have been summarized in Table 2.

**Table 2:** HLD induced cancer along with target pathways in different human and animal models.

Target organ	Target pathway	Cancer	References
1. Liver	Activation of Lymphotoxin-β receptor, lymphocytes, canonical NF-kB signaling pathway.	Hepatocellular carcinoma	[217]
2. Mammary gland	TGF-β1/SMAD3/miR-130 negative-feedback loop via down regulation of miR-140.	Breast cancer	[218]
3. Stomach	Activation of β-catenin, leptin and PI3K signaling pathway.	Gastric cancer	[161,219]
4. Intestine	Activation of PPARδ/β-catenin, inhibition of nuclear bile acid activated receptor (BAR) and farnesoid X receptor (FXR) signaling pathway.	Intestinal cancer	[220,221,222,223,224]
5. Pancreas	i. Activation of CCK-receptor pathway, signal transducer and activator of transcription-3 (STAT3), Kras and its downstream pathways including phospho-ERK, TNF-1 signaling. ii. Mutation in PI3K/Akt/mTORC1 pathway.	Pancreatic cancer	i. [225,226,227,228] ii. [226,229]
6. Prostate	i. Activation of IL6/pSTAT3 signaling, growth factor signaling (e.g., IGF-I/PI3K/AKT signaling cascade). ii. Pml and SREBP-supported altered and de novo lipogenesis. iii. Activation of inflammatory pathways via cytokine, chemokines storm and MCP-1/CCR2 pathway. iv. Modulation of endocrine pathways.	Prostate cancer	i.[230,231,232,233,234,235,236,237] ii.[234,238] iii.[239,240,241,234] iv.[242,243,234]

#### 4.8.1 Hepatocellular carcinoma

Nowadays, cancer has been marked as a significant life-threatening disease after cardiovascular complications. Earlier, we have already discussed the occurrence of NAFLD mediated systemic damage due to changes in food habits and consumption of HLD, which are enriched with saturated fatty acids like palmitic acid, lauric acid, and myristic acids hydrogenated fats mediated [3]. Here we have discussed the crosstalk between NASH and hepatocellular carcinoma; NAFLD advances to NASH, which further causes progression of fibrosis, cirrhosis, and hepatocellular carcinoma (HCC) [161].

Earlier study well demonstrated the synergistic impact of CD8^+^ T and natural killer T (NKT) cells in the progression of inflammation, fibrosis in the liver, which ultimately leads to the development of NASH [177]. A recent experiment was conducted to evaluate the underlying cause of the conversion of NASH to HCC, and the result suggests that long-term fat-enriched but choline-deficient diets developed NASH, and thereafter NASH mediated HCC; primarily a T cell-derived co-stimulatory ligand LIGHT secreted from the NKT cells, which, ultimately developed steatosis. Moreover, LIGHT binds to the hepatocellular activation of the lymphotoxin-β receptor (LTβR) induced lipid uptake by NKT cells. Furthermore, the molecular mechanism behind the transition of NASH to HCC is assisted by LTβR and classical NF-kB signaling pathways of the liver [217]. So, it can be hypothesized that HLD induced HCC was developed by the activation of the lymphotoxin-β receptor, lymphocytes, and canonical NF-kB signaling cascades.

#### 4.8.2 Malignancy of the mammary gland

Adipose tissue of the breast of humans is composed of a diverse number of cell populations such as mature white adipocytes, immune cells, multipotent mesenchymal stem cells, committed progenitor cells, fibroblasts, and endothelial cells. Mesenchymal stem cells can be transformed into heterogeneous cells like myofibroblasts, fibroblasts, and adipocytes by influencing foreign stimulus [244]. The microenvironment of adipose tissue is a positive contributor to breast cancer [245]. Seo et al. [246] demonstrated that the development of malignancy of the mammary gland and remodeling of the fibrotic microenvironment is influenced by obesity induced differentiated myofibroblast in the adipose tissue of the mammary gland [246]. In addition, HLD provokes the activation of TGF-β1 signals followed by stimulation of SMAD3, which ultimately reduces the expression of microRNA (miR)-140 after binding on it. Moreover, inhibition of miR-140 causes up-regulation of the TGF-β1/SMAD3 signaling pathway with decreased expression of miR-140, which further leads to differentiation of myofibroblast. Here, the TGF-β1/SMAD3/miR-130 negative-feedback loop plays a crucial role in a higher rate of differentiation of myofibroblast with reduced expression of miR-140 in fat tissue mammary gland due to consumption of lipid-enriched diets [218]. It has also been found from the earlier studies that the occurrence and metastasis of malignancy in the mammary gland are positively linked with myofibroblasts, and myofibroblasts induced a small-scale fibrotic environment [218,246]. So, it can be speculated that high lipid diets induced TGF-β1/SMAD3/miR-130 negative-feedback loop dependent higher risk of malignancy in mammary gland occurred via the reduced expression of miR-140 mediated differentiation of myofibroblast in adipose tissue of the mammary gland of human.

#### 4.8.3 Malignancy in the stomach

Stomach or gastric cancer progresses slowly compared to other cancers. Recently, researchers have identified some types of malignancies arising from the mucous membrane of the stomach, which are directly associated with the intake of HLD [161]. Moreover, very little information is available about the mechanism of dietary lipids-induced abnormal growth of cells. Therefore, cancer in the stomach stimulates the cellular functions of the stomach’s mucous membrane. Earlier studies well documented that the administration of lipid-enriched diet mediated deposition of lipid droplets in the gastric pits leads to abnormal cellular growth in the stomach’s mucous membrane in the murine model. Upregulation of calnexin, lysosome-associated membrane protein 2 (LAMP2), Golgi membrane (GM) 130, and cytochrome c oxidase (COX) subunit 4 in the mucous membrane layer of the stomach altered the homeostasis of the organelle due to abnormal accumulation of lipids on it [219].

In the more susceptible area of malignant growth in the stomach’s mucous membrane, the accumulation of lipids ultimately altered the homeostasis of the organelle. Activation of the β-catenin pathway provoked gastric mucosal-cancer development via the influence of the division of stem cells by lipid-enriched diet. Therefore, it can be observed that the β-catenin way is being regulated by the leptin signaling pathway [161,219]. Therefore, it can be speculated that β-catenin, leptin, and PI3K signaling pathways are associated with stomach cancer by consuming HLD.

#### 4.8.4 Intestinal Cancer

Cancer of the small intestine is a sporadic form of cancer compared to malignancy in the large intestine. There was accumulating evidence well documented about the relationship between HLD and cancer in the intestine [151,161]. A diet consisting of 60 % fat led to the rapid acquisition of cancerous properties by normal intestinal cells [220] well corroborated with earlier study [247]. Schulz et al. [247] stated that disturbances in a group of the bacteria in the gut in K-ras^G12Dint^ murine model without obesity promote intestinal tumors’ development [247]. The result of malignant growth by the daily intake of HLD occurred in the intestine directly or indirectly via shortening of the villi with a higher depth of the crypt of the intestine, the disturbed ratio of the intestinal stem cells and Paneth cell populations, augmentation of stem cells of the intestine without the Paneth cell-mediated signals and the formation of organoids in the intestine by progenitor stem cells [220,221]. However, there are two crucial pathways by which HLD induced a higher chance of occurrence of intestinal cancer; either by activating the PPARδ/β-catenin pathway via enhancing the unique differentiating character of the stem cells from normal cells and progression of malignant growth in progenitor intestinal cells [220] or by inhibition of the nuclear bile acid-activated receptor (BAR), farnesoid X receptor (FXR) signaling by reducing bile acid and actively participated in the proliferation of the colonic epithelial cells [221]. Simultaneously, some other reports suggested that there is an interconnection between the HLD provoked the activation of PPARδ signaling and beginning as well as the development of colon or rectal cancer [[222], [223], [224]]. Further investigations are required to address how high lipid diet-induced inhibition of FXR and activation of PPARδ signaling cascade.

#### 4.8.5 Pancreatic cancer

Cancer in the pancreas can occur on an exocrine or endocrine part, spread over the total pancreatic tissue. The duodenum secreted trophic peptide cholecystokinin (CCK) in response to high dietary intake of lipids enriched foods. In an earlier study, it has also been shown that high dietary intake of lipids; mainly saturated fatty acids and CCK are positively associated with malignancy in the pancreas [225], Some recent reports suggested that overweight further provoke malignancy [248,249]) by triggering the levels of insulin, insulin-like growth factor-1 (IGF-1), estrogen, which altered the level of leptin, visfatin, adiponectin, and disturbed intestinal mthe icrobiome by low-grade inflammation and immune modulation [225]. Moreover, inflammation plays a significant role in developing pancreatic cancer in obese animal models [227,250,251]. Nadella et al. [225] well documented that high dietary intake of lipids with high content of saturated fatty acids stimulate pancreatic cancer via the activation of the CCK-receptor pathway by increasing the level of CCK in blood, advanced fibrosis, maintenance of tumor microenvironment, reducing the cluster of differentiation 8^+^ lymphocytes with downregulation of tumor suppressor genes leading to metastasis [225].

Exocrine pancreatic cancer (e.g., pancreatic ductal adenocarcinoma) has been associated with a daily intake of a high amount of HLD; there is a relationship between Kras mutation, oncogenesis, and inflammation [227]. High dietary lipid intake stimulates the inflammatory response and thereby a positive contributor to the activation of Kras [228]. In addition, crosstalk between pancreatic acinar cells and neoplastic lesion in the pancreas occurs via substitution of a single base in Kras; moreover, Philip et al. [227] further well documented that high lipid diet activated the Kras via COX2 followed by an inflammatory response in the pancreas with stromal fibrosis, neoplastic lesion in the pancreas and pancreatic ductal adenocarcinoma from normal acinar cells of the pancreas [227]. Therefore, inactivated stellate cell in the pancreas further activates HLD induced increased expression of prolyl 4-hydroxylase, fibronectin, alpha polypeptide II, cadherin 11, alpha-smooth muscle actin via the Kras activation [226,227,252,253]. Signal transducer and activator of transcription-3 (STAT3) constitute a significant controller of the development of carcinogenesis in the pancreas. High dietary lipid consumption increased the phosphorylation of STAT3 [226]. Here, HLD contributed to fibro-inflammatory responses in the pancreas.

Oncogenic activated Kras promotes downstream stimulation of COX2, phosphor-ERK, and macrophage infiltration in the area surrounded by the pancreas’ neoplastic lesion leading to the motivation of the formation of pancreatic intraepithelial neoplasia [227]. Therefore, inflammatory response, involvement of pro-inflammatory mediators, and fibrosis in the pancreas developed via COX2 mediated positive feed-forward loop, which is regulated by the activity of Kras. Earlier investigation proposed that advancement of HLD induced intraepithelial neoplasia in the pancreas progressed by TNF-α via TNF-1 signaling [228]. Moreover, accumulating shreds of evidence suggested that pancreatic ductal adenocarcinoma developed via the stimulation of the anti-apoptotic, pro-inflammatory transcription factor NF-kB by the up-regulation of pro-tumorigenic multifunctional protein p62, a classical receptor of autophagy [226,[254], [255], [256]]. Therefore, it can be postulated that high lipid diets are responsible for pancreatic malignancy by inflammation and uncontrolled autophagy. Another significant observation of Chang et al. [226​] revealed the occurrences of pancreatic carcinoma depend on gender (i.e., male > female) [226].

It can be speculated that there are several inflammatory mediators which regulate the activity of Ras via positive feedback loop; higher incidence of pancreatic ductal adenocarcinoma developed through the mutation of oncogenic Ras in the cells of the pancreas by consumption of dietary lipids in high amount, but in average healthy person oncogenic Kras is present [227]. Therefore, it can be conjectured that a higher intake of dietary lipid-induced inefficient autophagy and inflammation with a higher risk of development of neoplasia in the pancreas, pancreatic ductal adenocarcinoma via downward stimulation of COX2, phosphor-ERK, macrophage infiltration. There is a relationship between pancreatic ductal adenocarcinoma and insulin signaling pathway via the modification of genes by high calorie contained high lipid diet [229]. Further, to justify the correlation between HLD and pancreatic carcinoma, some researchers identified another signaling cascade involved via several mutations in PI3K/Akt/mTORC1 pathway causes alteration of amino acids, which ultimately leads to modification of polarity, pH, and hydropathy to such signaling proteins [226,229].

#### 4.8.6 Prostate cancer

Dietary patterns and prostate cancer are linked with each other. Previously, researchers and scientists demonstrated that the consumption of lipids enriched food enhances tumorigenesis of prostate cancer by the influence of various cytokines [231]. Moreover, accumulating pieces of evidence well demonstrated that high dietary lipid up-regulated the expression of IL-6 and IL-13 in prostatic tissue and escalated population myeloid-derived suppressor cells in a murine model [231,257]. Further, the development of the tumor was triggered by IL-6 signaling through the phosphorylation of a signal transducer and activator of transcription 3 (pSTAT3) [230]; additionally, IL-13 stimulates macrophage polarization to alternatively activated macrophage (M2) [258]. Hayashi et al. [231] also demonstrated that the local macrophages secreted IL-6 and accelerated the growth of the prostate tumor via pSTAT3 with enhanced the population of myeloid-derived suppressor cells in the microenvironment of pro-tumors [231]. Myeloid-derived suppressor cells act as a suppressor of multiple immune effectors and T cells [259]. It can be postulated that consumption of HLD leads to higher production of myeloid-derived suppressor cells, increased ratio of alternatively activated macrophage (M2) to classically activated macrophage (M1), and thereby progression of prostate cancer via the IL6/pSTAT3 signaling cascade in such patients with or without obesity [231].

Increased intakes of HLD induced obesity and increase the level of insulin are directly linked with higher insulin-like growth factor-I in circulation which is a critical factor for the development of different cancers, including prostate cancer [234,249] via modulation of IGF-I receptor (IGF-IR) signaling [232]. Further, activation of IGF-I/IGF-IR axis has also been associated with HLD-induced obesity-mediated cancer [233]. Moreover, inactivation of phosphatase and tensin homolog (PTEN) either by deletion or mutation promoted oncogenic activation of PI3K-AKT signaling to boost the metastasis [234]. Previously, researchers also reported the HLD-induced prostate cancer could be developed and progressed via IGF-I/PI3K/AKT signaling pathway [[235], [236], [237]]. Therefore, it can be speculated that IGF-I/PI3K/AKT signaling is the primary target pathway of HLD-induced prostate cancer initiation and development. However, there is no such impact of various sources of fats on the IGF/insulin axis. Therefore, it can be suggested that more studies are required for the effects of different sources of fats in HLD induced development of prostate cancer.

The dietary intake of high amounts of lipid-induced prostate cancer via up-regulation of the human genome encodes seven elongase enzymes (ELOLV7) [260]. However, up-regulation of scavenger receptor class B, type I (SR-BI), and angiogenesis leads to metastasis of tumor by intake of high lipids containing Western-style diet [234]. In addition, Huang et al. [​^261^​] proposed that the reduced level of FASN was inversely associated with high dietary lipids induced prostate cancer [261]. Moreover, alteration of lipogenesis influenced prostate cancer’s metastasis by regular intake of Western-diet as a high dietary lipid source [234]. Promyelocytic leukemia protein (Pml) inactivation and reactivated mitogen-activated protein kinases (MAPK) leads to lethal metastatic tumors from indolent PTEN-null prostate tumors followed by overactivation of sterol responsive element-binding proteins (SREBP) with altered total lipid profiles. Further regulator of the lipogenic gene SREBP stimulated by high intake of dietary lipids leads to fat deposition in non-adipose tissue [234,238]. Therefore, it can be postulated that a higher rate of progression of prostate cancer may be correlated with Pml, SREBP-linked alteration of lipogenesis, and de novo lipogenesis. Furthermore, there is no such evidence about high dietary intake of lipids-induced prostate cancer due to accumulation of exogenous fat and endogenous alterations of lipid profiles.

The high amount of dietary lipids is associated with an obesity-induced inflammatory response in adipose tissue via cytokines’ secretion [239]. Higher levels of monocyte chemoattractant protein (MCP)-1, MCP-5, tissue inhibitors of matrix metalloproteinases (TIMP)-1, IL-16, chemokine (C-C motif) ligand (CCL)12, chemokine (C-X-C motif) ligand (CXCL)1, CXCL10, and CXCL13 in serum are associated with intake of HLD due to the up-regulation of the cluster of differentiation (CD)3, CD45, forkhead box protein P3 (FoxP3), MCP-1, IL-6, and TNF-α [240]. Additionally, Huang et al. [241] well documented that infiltration of macrophages was strongly correlated with HLD induced progression of the prostate tumor of humans via MCP-1/CCR2 pathway [241]. Moreover, the palmitic acid in the HLD [3] activated macrophage inhibitory cytokine 1 (MIC-1), transforming growth factor-b, which is also a contributor to prostate cancer [262]. In some rodent models, higher expression of FABP4 and interleukin-8 also triggered the occurrence of lipids enriched diet-induced prostate cancer [128]. Furthermore, TNF-like weak inducer of apoptosis (TWEAK), CCL3, CCL4, and CCL5 like cytokines and chemokines are also associated with prostate cancer development the daily consumption of fatty food [234]. High dietary lipid-induced hypertrophy of adipocytes promoted Cav-1 secretion for the development of carcinogenesis in the prostate [263,264]. It can be assumed that carcinogenesis in the prostate developed via the activation of cytokines from adipocytes, inflammation, and infiltration of immune cells into tumors due to high dietary intakes of fatty foods.

Lipid enriched diet enhanced the progression of prostate cancer via dysregulation of the hormonal mechanism [242,243]. In the process of carcinogenesis in the prostate, sex hormones play a significant role [234]. Earlier reports well documented that a high lipid-containing diet without omega-3 fatty acids or a quantitatively low amount of omega-3 fatty acids altered the hormonal profiles within the tumor, which are positively correlated with the maturation and advancement of malignant tumor [234,265]. Massillo et al. [266​] proposed that high estradiol in serum contributed to fast growth of tumor in transgenic adenocarcinoma mouse prostate (TRAMP)-C1 allograft model via the up-regulation of transcription of cytochrome P450 aromatase (CYP19A) which further stimulated the conversion of androgen to estrogens; the transcription was regulated by intratumoral C-terminal-binding protein 1 (CtBP1) in lipids enriched diet received a murine model with rapid growth of neuroendocrine tumor model (e.g., TRAMP-C1 allograft tumor model) [266]. Another report well documented that depletion of CtBP1 resulted in small and slow growth of tumor in an androgen-independent model (e.g., PC-3 xenografts model) [267]; therefore, it can be suggested that CtBP-1 plays a significant role in the modulation of hormones within the tumor by helping of transcriptional factors and leads to the development of high dietary intakes of lipids induced carcinogenesis in the prostate.

## 5 Alarming impact of savory tastant in HLD on human health

Nowadays, excessive use of MSG in HLD as ready-made foods increases rapidly due to the general public’s demand. There are various harmful effects of MSG and HLD independently in animal models and human beings. The present review hypothesizes that if MSG and HLD alone can induce toxic effects on different animal models as a human trial, then the ready-made foods containing these ingredients may also produce a deadly impact on human health. However, there is very little information on the combined effect of MSG and HLD on OS-related metabolic disorders. In normal homeostasis, for different biochemical pathways, intracellular signaling, defense against microorganisms, and cellular functions, low ROS levels are essential. The higher production of ROS altered the equilibrium between the ratio of the oxidants or pro-oxidants and antioxidants agents, which ultimately prompt lipid peroxidation and depleting both enzymatic and non-enzymatic antioxidant cellular reserves, causing tissue damage and programmed cell death [268]. Some small scale preliminary studies suggested that flavor-enhancing chemicals like MSG and hydrogenated fats in diet or, more precisely, ready-made foods are the dietary operators of altered expression of a gene that developed disease with a term of phenotype in psoriasis and hypertensive human [[269], [270], [271], [272]].

Pedram et al. [273] reported the relationship between food addiction and severity of obesity; body composition is higher in women than men [273]. Some earlier reports demonstrated that regular consumption of protein and fat-based foods of diet is considered as food addiction as per Yale Food Addiction Scale (YFAS) in individuals [273,274]; individuals with obesity also consume a more significant amount of fat is also considered as food addicted as per the criteria of YFAS [275]. Another study demonstrated that the ubiquity of food addiction was about 38.3 % higher in the population with obese people as compared to healthy people [276]. Obregón et al. [277] also stated that about 30 % of the prevalence of food-addicted people in an area is marked as obese [277]. From these shreds of evidence, we can say that food addiction and obesity are strongly correlated with each other, i.e., more food-addicted people in an area; more will be the prevalence of obesity prevalence. A report by Pongking et al. [​^82^​] showed that MSG along with HLD induced kidney damage by altered gut microbiota, increased p-cresol sulfate levels in urine, kidney injury molecule (KIM)-1, and high mobility group box protein 1 (HMGB-1) expression in the kidney; it is also a risk factor for CKD [82]. In children and adolescents, fast-food consumption like Sausage, French fries, and pizza are positively linked with metabolic syndromes with the obese abdomen, increased triglycerides that may develop diabetes, and cardiovascular disorders like deadly disease [278]. Nowadays, metabolic syndromes are spreading quickly worldwide in both children and adolescents, and it becomes a significant health concern [279]. The flavor-enhancing high lipid diet as fast-food has several unique features like high glycemic load, palatability, primeval preferences of taste, immoderate size of the portion, more important daily energy requirements of healthy individuals [278]. Tavares et al. [​^280^​] demonstrated in Brazilian adolescents that increase consumption of ultra-processed foods are directly linked with metabolic syndromes [280]; moreover, higher consumption of ready-made foods are also linked with increased risk of IR [278]. Additionally, it has been proved from the earlier studies that regular consumption of such kinds of food increased the risk of obesity and cardio-metabolic disorders [[281], [282], [283]]. Kelishadi et al. [281] reported that overweight and obese children consumed 2.7 times of fast-foods per week as compared to normal children who consumed 1.2 times per week in the Isfahaninan population [281]. The nationwide representative study demonstrated fast-food intake is also positively correlated with obesity in children and adolescents of America [283]. An earlier report stated a significant correlation between the consumption frequencies of fast-foods with hydrogenated fats, cheese puffs, chips, and altered lipid profile levels in urban and rural children of Iran [282].

We already reported that different food industries, cafeteria, restaurants add a high amount of MSG for the strategy of flavor enhancement in the fast-food formulation, and such kinds of food have a high glycemic index with energy density because of its high amounts of salts in the form of MSG instead of common salts like sodium chloride, trans-fatty acids, saturated fatty acids, hydrogenated fats with low levels of dietary fiber [3,51,278]. The permitted use of trans-fatty acid is significantly less in quantity with lower than 10 % calories from saturated fats in Iran; on the contrary, they have used about 23.6%–30.6% of trans-fatty acids in the total content of fatty acids. Over the last half-century, in fast-food restaurants, the portion sizes of burgers, fried potatoes, pizzas as fast-food increased by two to five folds which is another major factor. An earlier investigation on the content of fatty acids in fast-food reported that in French fries, fried chicken contains about 41 g–74 g of total fat, 0.3 g–24 g of trans-fat used by renowned food-chain restaurants in the World [278].

Saturated and trans-fatty acids in the form of HLD intake increased the expression and secretion of both IL-6 and CRP, respectively [284,285], which altered the normal metabolic homeostasis of the body and appeared to be a key factor for different diseases such as glucose intolerance, hypertension, insulin resistance, hyperlipidemia as a part of metabolic syndrome [3,286].

On the other hand, another study demonstrated that prolonged intake of flavor-enhancing chemicals like MSG-rich diet in a healthy population attenuates the umami taste sensation and simultaneously diminishes the appetite center for savory foods selectively in females [287].

Earlier studies reported that strong gustatory stimuli impaired the satisfaction of gustatory response of salt, sweet and fat or transferred fondness to the diet-enriched with such taste stimuli [[288], [289], [290]]. Taste receptor type 1 member 1 (T1R1) or T1R3 subunits are responsible for sweet, savory taste sensations and influenced by this receptor [291]. Further, some other studies demonstrated that regular intake of MSG in diet-induced down-regulation of the T1Rs reduced the sense of savory impulses [287,292]. Wang et al. [293] proposed male also eat more protein as well as MSG-enriched foods than females [293] but not the different properties with the sense of gustatory impulse of umami; hence, it can be speculated that males are more prone to MSG induced anomaly than female and there is a complex association between the satiation, hunger and savory taste in human.

Due to the universal demand of fast-food and restaurants in developing areas were converted to a more famous site because of their advertisement. Regular consumption of flavor-enhancing high lipid diet in the form of ready-made foods all over the world is positively associated with nutrition-related non-communicable diseases (NR-NCD). Nowadays, NR-NCD becomes a global burden, affecting both the wealthy and developing countries. Further investigation on cultural factors induced rapidly increasing NR-NCD can only give insight into some of the world's most unfavorable problems [278]. A recent study by Banerjee et al. (2020) suggested that MSG mixed HLD caused NAFLD mediated systemic damage via generation of ROS with altered redox-homeostasis and pro-inflammatory/anti-inflammatory factors, which ultimately leads to apoptosis possibly through p53/p21 and mitochondrial caspase-mediated signaling [14]. A recent report proposed that flavor enhancing diet generates ROS and alters the redox homeostasis that indirectly stimulate the severe acute respiratory syndrome coronavirus 2 (SARS-CoV-2) mediated infection causes novel coronavirus (COVID-19) outbreaks [294].

Higher intake of flavor-enhancing high lipid diet in the form of ready-made foods has an undesirable impact on the occurrence of metabolic syndromes, hypertriglyceridemia, obesity, the cardio-metabolic risk for both children and adolescents. Further, health care strategies are required for the prevention of such anomalous conditions.

## 6 Alarming message of the uses of MH in regular diet and possible strategy of management to fight against MH induced anomalous situations

**Fig. 4:**
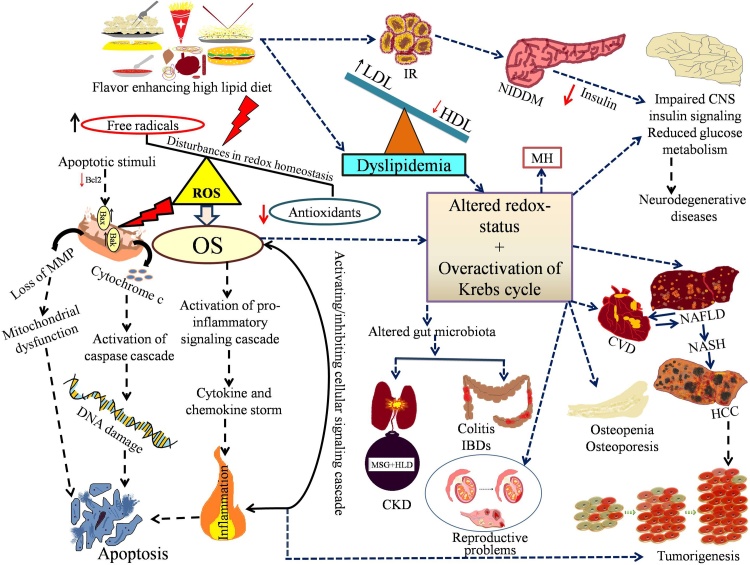
Hypothetical target pathway of the deleterious impact of savory tastant in HLD as a part of ready-made foods with their alarming impact (the black upward arrow indicates increase and red downward arrow indicates decrease).

Fig. 4 shows the hypothetical target pathway of the harmful impact of MSG mixed HLD as a part of ready-made foods with their alarming implications. The random use of MSG in HLD and frequent intake of MH knowingly by hungry-busy-working people at regular intervals in quantitatively higher levels ultimately leads to a warning impact on human health. Earlier studies demonstrated that about 1.5 grams–10 grams of MSG had been used in daily diets in Japan, Korea. Therefore, immense and frequent use of MSG combined with HLD is an excellent warning for public health. Moreover, no company shows how much they use MSG as a seasoning substance in their foods and meals or whether they use it at all, but they do use it. Because there is no such law to enhance the taste of food by adding unhealthy chemicals, some restaurants are using MSG instead of salt for improving the taste of specific foods, and surprisingly, they have proudly declared that they have not used food additives in their restaurant or the foods [9]. Consumption of "junk food" as a heavy diet may cause obesity resulting in the emergence of movements towards public health consciousness and a ban on advertising of such commodities [296]. The addiction to a particular food or junk foods with high amounts of lipid is strongly correlated with food-related health problems in developing countries. So food addiction is considered a phenomenon for decreasing health problems that mainly arise from foods [274]. Consequently, food addiction is related to energy-rich food consumption and obesity, primarily abdominal fat accumulation. Since food addiction is predominantly influenced and provoked by compulsive behavior and emotional and environmental factors such as stress, socio-cultural status, and chronic characteristics, these factors should be considered in future studies. Future randomized controlled research would also identify elements, combinations, and concentrations of ingredients that are potentially addictive. Furthermore, the improvement of nutrition knowledge and the promotion of consciousness in the general population regarding the harmful use of food additives in ready-made foods might be essential for a healthy lifestyle. In this context, the present review identifies alarming points about the detrimental impact of MSG and high lipid diet independently and combined as a part of ready-made foods, which may lead to systemic injury and various systemic anomalies. It can be suggested that we have to prepare the diet by reducing the use of saturated fatty acids, trans-fat, hydrogenated fats in a regular diet [3]. Meals can be prepared by mixing natural antioxidants, vitamins from different sources such as fruits, vegetables, etc., having ethnopharmacological relevance; reducing or replacing ready-made foods with a high-fiber-enriched diet, which is also another good source of antioxidants without any side effects instead of taking a synthetic drug. This is an alternative approach to reduce the harmful effects of flavor-enhancing HLD in the human body. Additionally, it was believed that this type of foods generally does not exert any adverse effects when incorporated with a well-balanced diet [8]; therefore, there must be some factor(s) that blunted the deleterious impact of MSG mixed HLD as ready-made foods when taken with proper balance diet or the balanced may contain some natural antioxidant which can reduce these toxic effects. Hence, it can be speculated that taking a balanced diet should be another viable strategy of management to combat systemic anomalies.

Shreds of evidence indicate the reduction of the concentration of total cholesterol, LDL as well as overall blood lipid profiles by replacing the saturated fatty acids based HLD with monounsaturated fatty acids (MUFA), polyunsaturated fatty acids (PUFA), or carbohydrate-based diet [153,156]. Both PUFA and MUFA are less concerned with the disturbances in cardiovascular events [295]. In addition, some cohort and randomized trial studies also demonstrated that lower consumption of saturated fatty acids in HLD is linked with the decrease in chances of cardiovascular disorder or replaced by PUFA. Moreover, replacement of the saturated fatty acids by plant-derived PUFA attenuates the risk of cardiovascular diseases in adults [153]. It can be suggested that as both PUFA and MUFA are beneficial for health, such researchers should study various effects of a mixture of these two, and whether it can be a viable strategy of management against the saturated fatty acids rich diet-induced anomalous situations or not which could be an alternative approach of using these mixtures by eliminating the saturated fatty acids from a regular diet. Additionally, plant-derived sterols also help reduce the dyslipidemic condition by maintaining LDL ratio to HDL. Therefore, phytosterol-containing food products can be used as therapeutic dietary options for reducing the risk of hypercholesterolemia and atherosclerosis. Phytosterol, like 24-epibrassinolide, acts as an antioxidant and protects neuronal cells from 1-methyl-4-phenylpyridinium (MPP)-induced OS and apoptosis. β-sitosterol acts as an antidiabetic, antioxidant drug; moreover, phytosterol also decreases NAFLD and NASH’s chance [188]. This review also suggests that researchers may investigate the ameliorative effect of β-sitosterol, other plant sterols, or bioactive compounds from the natural source of antioxidants on MSG mixed HLD induced changes, if any.

Another alternative strategy of management against the aforementioned anomalies can be attenuated by using specific inhibitors. Suppose we can track the target signaling pathway(s) by which flavor-enhancing high lipid diet exerted the detrimental effects. In that case, we can inhibit the signaling by using specific inhibitors without any adverse effects on the body. For example, statins are β-hydroxy β-methylglutaryl-CoA (HMG-CoA) reductase inhibitors. Some cholesterol absorption inhibitors reduce elevated hepatic markers enzymes and thereby lowered NAFLD occurrence by inhibiting LXRα-SREBP-1c pathway. Therefore, a reliable therapeutic strategy for patients with NAFLD is cholesterol metabolism regulation under high lipid diet conditions [188].

A recent study by Banerjee et al. [14] suggested that MSG mixed HLD induced systemic damage could be suppressed by naturally occurring plant *Coccinia* grandis [14]; ethanol extract of *Coccinia grandis* leaves have a great source of *in vivo* antioxidant and free radical neutralizing activity [58] due to the presence of so many bioactive compounds with different therapeutic properties to reduce the generation of ROS in the body by MSG mixed HLD [14]. Moreover, it also beneficially attenuate metabolic disturbances, dyslipidemic condition, inflammation, programmed cell death, and combat against the effects of co-administration of monosodium glutamate mixed high lipid diet-induced non-alcoholic fatty liver disease associated systemic anomalies [14].

Hence, it can be further suggested that as an alternative to synthetic drugs with different side effects, it seems to be the viable strategy of management to use naturally occurring plant extract (i.e., *Coccinia* grandis) with minimal or without side effects against flavor-enhancing HLD induced damage in the body. Furthermore, it can be postulated that inhibition of the target pathway or use of inhibitors or blockers to the targeted pathway by which MSG or HLD, or MH exert their adverse effects on animals and the human body may be another alternative molecular approach of therapies.

## 7 Conclusions

The present review warns about MSG or HLD alone, or MSG mixed HLD as ready-made foods. Their adverse effects on different systems enlighten the dietary risk factors that may be considered to make awareness among ordinary people about their food habits. This initiative is very much needed to cause the threat of metabolic disorder imperative as well as systemic damage. So, as the source of both trans-fatty acids, saturated fatty acids in partially hydrogenated or hydrogenated and MSG as flavor enhancers have adverse health effects. To combat these negative effects, we should modify our diet with good sources of antioxidants, high-dietary fiber with PUFA and MUFA as an alternative source of saturated fatty acids, trans-fatty acids, and hydrogenated fats in preparation of dishes. Moreover, MSG and HLD exerted deleterious effects by modulating different signaling cascades to cause dyslipidemia via altered LDL/HDL and leptin/adiponectin ratio, OS via generation of ROS with altered redox-homeostasis, inflammatory response via altering the balance of pro-inflammatory/anti-inflammatory factors, apoptosis via stimulating apoptotic mediator and thereby systemic damages; different animal models are now being developed to understand the pathways involved in systemic anomalies and cancer by MSG/HLD; however, further experiments are needed to confirm whether these signal pathways are physiologically valid for humans or not and at the same time, it is very important to find the transcription factors and genes involved in this systemic injury or even cancer.

## Grants, sponsors, and funding sources

Not applicable.

## Declaration of Competing Interest

The authors report no declarations of interest.
